# Visual outcomes and their association with grey and white matter microstructure in adults born preterm with very low birth weight

**DOI:** 10.1038/s41598-024-52836-4

**Published:** 2024-02-01

**Authors:** Sigrid Hegna Ingvaldsen, Anna Perregaard Jørgensen, Arnstein Grøtting, Trond Sand, Live Eikenes, Asta K. Håberg, Marit S. Indredavik, Stian Lydersen, Dordi Austeng, Tora Sund Morken, Kari Anne I. Evensen

**Affiliations:** 1 Department of Neuromedicine and Movement Science, NTNU Norwegian University of Science and Technology, Trondheim, Norway; 2 Department of Ophthalmology, St. Olav Hospital, Trondheim University Hospital, Trondheim, Norway; 3 Department of Neurology and Clinical Neurophysiology, St. Olavs Hospital, Trondheim University Hospital, Trondheim, Norway; 4 NorHEAD - Norwegian Centre for Headache Research, Department of Neuromedicine and Movement Science, NTNU Norwegian University of Science and Technology, Trondheim, Norway; 5 Department of Radiology and Nuclear Medicine, MR-Center, Trondheim University Hospital, Trondheim, Norway; 6 Department of Clinical and Molecular Medicine, Norwegian University of Science and Technology, Trondheim, Norway; 7 Regional Centre for Child and Youth Mental Health and Child Welfare, Department of Mental Health, Faculty of Medicine and Health Sciences, Norwegian University of Science and Technology, Trondheim, Norway; 8 Department of Physiotherapy, Oslo Metropolitan University, Oslo, Norway

**Keywords:** Visual system, Magnetic resonance imaging

## Abstract

Individuals born with very low birth weight (VLBW; < 1500 g) have a higher risk of reduced visual function and brain alterations. In a longitudinal cohort study, we assessed differences in visual outcomes and diffusion metrics from diffusion tensor imaging (DTI) at 3 tesla in the visual white matter pathway and primary visual cortex at age 26 in VLBW adults versus controls and explored whether DTI metrics at 26 years was associated with visual outcomes at 32 years. Thirty-three VLBW adults and 50 term-born controls was included in the study. Visual outcomes included best corrected visual acuity, contrast sensitivity, P100 latency, and retinal nerve fibre layer thickness. Mean diffusivity, axial diffusivity, radial diffusivity, and fractional anisotropy was extracted from seven regions of interest in the visual pathway: splenium, genu, and body of corpus callosum, optic radiations, lateral geniculate nucleus, inferior-fronto occipital fasciculus, and primary visual cortex. On average the VLBW group had lower contrast sensitivity, a thicker retinal nerve fibre layer and higher axial diffusivity and radial diffusivity in genu of corpus callosum and higher radial diffusivity in optic radiations than the control group. Higher fractional anisotropy in corpus callosum areas were associated with better visual function in the VLBW group but not the control group.

## Introduction

Preterm birth with very low birth weight (VLBW, birth weight < 1500 g) increases the risk of several neurodevelopmental difficulties later in life, including visual problems^1^. Follow-up studies of VLBW individuals have revealed reduced visual acuity compared to their peers from childhood to adulthood, even without a history of ophthalmological or cerebral injury at birth^2,3^. Additionally, contrast sensitivity (CS) has shown to be reduced in low and high spatial frequencies among children and adolescents born with VLBW without neurological complications^3,4^.

Information from visual stimuli is conveyed by the ganglion cells in the retinal nerve fibre layer (RNFL) via the optic nerve, where the information from both eyes merges at the optic chiasm^5^. A thick layer of ganglion cells and fast conduction of signals via the optic nerve is, therefore, essential for normal visual functioning. Compared with controls, a thicker central RNFL has previously been found in a group of young adults born extremely preterm^6^. Moreover, pre-schoolers born preterm with birth weight < 2500 g displayed slower pattern-reversed visual evoked potentials (PR-VEPs) responses than their normal birthweight peers^7^ indicating slower conduction of visual signals. Reduced visual outcomes in those born preterm might not be solely due to vascular sequela caused by retinopathy of prematurity (ROP). Instead, visuopathy of prematurity (VOP), an overarching inflammation-initiated framework including perinatal brain injury and neovascularization of the retina, might be a better explanation^8^. Indeed, those born preterm with VLBW have a substantially elevated risk of perinatal brain injury^9^, especially in white matter (WM) microstructure^10^.

Both grey matter (GM) and WM microstructure can be investigated with diffusion tensor imaging (DTI), a magnetic resonance imaging (MRI) technique^11^. Perinatal brain injury is usually accompanied by higher mean diffusivity (MD) and lower fractional anisotropy (FA) in WM^12^. Higher MD characterizes increased overall diffusivity, reflecting less cellular membranes and/or packing in a particular voxel^13,14^. Lower FA is considered a proxy for disorganization or decreased WM microstructural integrity^14^. However, FA vary depending on the WM tract and the crossing fibres of a region. Therefore, a difference in FA between groups only expresses a difference in the orientation-dependent microstructure in a voxel and/or region. To further explore whether a group difference in FA is related to the degree of myelination or axon density, radial diffusivity (RD) and axial diffusivity (AD) metrics need to be considered^15^. AD is considered to represent/is a proxy for diffusion parallel to the WM tracts, while RD, is a proxy for diffusion perpendicular to the WM tracts. These additional DTI derived biomarkers help interpret whether a lower FA and higher MD are related to axonal injury and poor fibre organization (lower AD) or poor myelination and axon packing (higher RD) in individuals born with VLBW^16,17^.

This study is part of the NTNU Low Birth Weight in a Lifetime Perspective (NTNU LBW Life) study, a longitudinal cohort study of individuals born preterm with VLBW and a term-born control group in Central Norway that have been followed from infancy to adulthood^1^. A WM microstructure pattern of DTI metrics with lower FA and higher MD has been shown in previous assessments of this cohort in adolescence^18^ young adulthood (18–22 years)^12^ and adulthood (25–28 years)^13^. In adolescents, Lindqvist et al.^19^ reported an association between lower visual acuity and lower FA in the splenium and body of corpus callosum (CC) due to an association of lower visual acuity with higher RD, and several smaller clusters in the frontal WM microstructure in the VLBW group from DTI data acquired at 1.5 tesla (T). This study only explored DTI data in corpus callosum and frontal areas with less detailed measures of visual acuity and contrast sensitivity. Whether the same association of altered WM microstructure pattern with visual problems involves other regions in the visual pathway and other measures of visual function and structure is unknown.

Therefore, this study aimed to assess whether visual function, retinal structure, and visual pathway function in the NTNU LBW Life study differ between the VLBW and control group at 32 years. Furthermore, we explored whether the visual outcomes were associated with the participants' WM and cortical GM microstructure at 26 years. Our objectives were (1) to estimate whether there were differences in BCVA, CS, P100 latency from patterned-reversed visual evoked potentials, RNFL thickness, and cortical GM and WM microstructure from DTI data acquired at 3 T in seven predefined regions of interest (ROIs) along the visual pathways between the VLBW and control group, and (2) to explore whether FA in WM ROIs and MD in primary visual cortex at 26 years was associated with visual outcomes at 32 years in the VLBW group.

## Results

### Clinical characteristics

Clinical characteristics of the VLBW and the control group are presented in Table 1. Birth weight and gestational age differed by definition between the groups. Head circumference at birth and Apgar score after 1 and 5 min were lower in the VLBW group. In addition, three participants in the VLBW group had IVH (grade 1–4) and/or PVL.

**Table 1:** Descriptive statistics for the VLBW group and the control group.

	VLBW (n = 33)		Controls (n = 50)	
	Mean	(SD)	Mean	(SD)
Maternal age^a^	30.0	(5.3)	31.3	(4.5)
Parental SES (1–5)^b^	3.3	(1.3)	3.9	(1.1)
Birth weight (grams)	1274.7	(203.6)	3736.1	(472.4)
Gestational age (weeks)	29.6	(2.8)	40.0	(1.2)
Head circumference at birth (cm)^c^	27.5	(2.2)	35.6	(1.1)
Apgar score after 1 min^d^	7.1	(1.7)	8.9	(0.4)
Apgar score after 5 min^d^	8.8	(1.1)	9.9	(0.4)
Age at brain MRI (26 years)	26.2	(0.7)	26.5	(0.4)
Age at clinical assessment (32 years)	32.4	(0.8)	32.5	(0.5)

	n	(%)	n	(%)
Males	13	(39.4)	21	(42.0)
Intraventricular haemorrhage (grade 1–4) and/or periventricular leukomalacia^e^	3	(9.1)	0	NA
Neurosensory impairments (yes)	4	(12.1)	0	NA

*MRI* magnetic resonance imaging, *NA* not applicable, *SD* standard deviation, *SES* socioeconomic status 1–5, where five is highest, *VLBW* very low birth weight.

^a^Data missing for one control participant.

^b^Data missing for two VLBW participants and eight control participants.

^c^Data missing for five VLBW participants and three control participants.

^d^Data missing for one VLBW participant and three control participants.

^e^Data missing for one VLBW participant.

### Visual outcomes

Table 2 shows mean differences between the VLBW and the control group on visual outcomes (BCVA, CS function, P100 latency, RNFL thickness). The VLBW group generally displayed a thicker central RNFL and lower CS function than the control group, with a mean difference of almost one CS threshold. Also, the VLBW group showed a mean P100 latency of 2.4 ms longer than the control group. The data distribution of the visual outcomes for VLBW and control participants are presented in Supplementary Figure B1 online. After adjusting the p-values for multiple comparisons with the Benjamin-Hochberg approach, the observed mean differences between the control and VLBW group were no longer statistically significant. There are some missing data from the PR-VEP assessment (P100 latency) and OCT assessment (RNFL thickness). The missing P100 latencies are due to movement artefacts leading to noise in the PR-VEPs which made in difficult to find a reliable P100 component. The missing OCT data is partially due to technical difficulties with the OCT machine. Also, we were not able to take a reliable OCT image of four of the participants due to movement artefacts.

**Table 2:** Mean (SD) of visual outcomes at 32 years and DTI metrics in ROIs at 26 years in the VLBW group compared with the control group.

	VLBW (n = 33)		Controls (n = 50)		Mean difference	95% CI	p-value	p-value^adj^
	Mean	(SD)	Mean	(SD)				
Visual outcomes								
BCVA	86.9	(3.7)	88.2	(4.0)	− 2.1	(− 4.9, 0.2)	.101	.245
CS function	5.1	(1.7)	6.0	(1.3)	− 0.8	(− 1.4, − 0.1)	.029	.195
P100 latency (ms)^a^	101.2	(5.6)	98.7	(4.7)	2.4	(0.04, 4.7)	.052	.217
RNFL thickness (µm)^b^	11.7	(1.7)	11.0	(2.3)	0.9	(0.05, 1.8)	.037	.195
FA/MD								
FA in genu (CC)	60.7 × 10^–2^	(3.0 × 10^–2^)	61.5 × 10^–2^	(2.3 × 10^–2^)	− 1.0 × 10^–2^	(− 2.5 × 10^–2^, 0.4 × 10^–2^)	.146	.245
FA in body (CC)	60.6 × 10^–2^	(4.3 × 10^–2^)	61.9 × 10^–2^	(2.7 × 10^–2^)	− 1.4 × 10^–2^	(− 3.5 × 10^–2^, 0.4 × 10^–2^)	.123	.245
FA in splenium (CC)	72.2 × 10^–2^	(4.8 × 10^–2^)	73.0 × 10^–2^	(2.5 × 10^–2^)	− 0.8 × 10^–2^	(− 2.9 × 10^–2^, 1.1 × 10^–2^)	.385	.544
FA in ORs	57.8 × 10^–2^	(3.5 × 10^–2^)	58.8 × 10^–2^	(3.3 × 10^–2^)	− 1.2 × 10^–2^	(− 2.9 × 10^–2^, 0.4 × 10^–2^)	.157	.245
FA in LGNs	35.9 × 10^–2^	(1.6 × 10^–2^)	36.6 × 10^–2^	(1.6 × 10^–2^)	− 0.7 × 10^–2^	(− 1.4 × 10^–2^, 0.1 × 10^–2^)	.062	.221
FA in IFOFs	52.1 × 10^–2^	(2.5 × 10^–2^)	52.2 × 10^–2^	(2.8 × 10^–2^)	− 0.3 × 10^–2^	(− 1.6 × 10^–2^, 1.0 × 10^–2^)	.658	.738
MD in V1 (mm^2^/s)	7.8 × 10^–4^	(0.37 × 10^–4^)	7.6 × 10^–4^	(0.64 × 10^–4^)	0.19 × 10^–4^	(− 0.047 × 10^–4^, 0.40 × 10^–4^)	.137	.245
AD (mm^2^/s)								
Genu (CC)	15.5 × 10^–4^	(0.93 × 10^–4^)	14.9 × 10^–4^	(1.0 × 10^–4^)	0.57 × 10^–4^	(0.19 × 10^–4^, 0.96 × 10^–4^)	.007	.175
Body (CC)	15.7 × 10^–4^	(0.65 × 10^–4^)	15.7 × 10^–4^	(1.2 × 10^–4^)	0.041 × 10^–4^	(− 0.36 × 10^–4^, 0.36 × 10^–4^)	.885	.885
Splenium (CC)	16.2 × 10^–4^	(0.74 × 10^–4^)	16.0 × 10^–4^	(1.1 × 10^–4^)	0.14 × 10^–4^	(− 0.27 × 10^–4^, 0.50 × 10^–4^)	.467	.584
ORs	12.6 × 10^–4^	(0.62 × 10^–4^)	12.4 × 10^–4^	(0.89 × 10^–4^)	0.26 × 10^–4^	(− 0.071 × 10^–4^, 0.54 × 10^–4^)	.128	.245
LGNs	12.3 × 10^–4^	(0.55 × 10^–4^)	12.4 × 10^–4^	(1.3 × 10^–4^)	− 0.077 × 10^–4^	(− 0.46 × 10^–4^, 0.21 × 10^–4^)	.679	.738
IFOFs	12.0 × 10^–4^	(0.54 × 10^–4^)	11.8 × 10^–4^	(0.89 × 10^–4^)	0.13 × 10^–4^	(− 0.19 × 10^–4^, 0.39 × 10^–4^)	.422	.555
V1	9.4 × 10^–4^	(0.39 × 10^–4^)	9.2 × 10^–4^	(0.72 × 10^–4^)	0.23 × 10^–4^	(− 0.041 × 10^–4^, 0.46 × 10^–4^)	.125	.245
RD (mm^2^/s)								
Genu (CC)	5.1 × 10^–4^	(0.71 × 10^–4^)	4.8 × 10^–4^	(0.65 × 10^–4^)	0.37 × 10^–4^	(0.09 × 10^–4^, 0.66 × 10^–4^)	.026	.195
Body (CC)	5.1 × 10^–4^	(0.66 × 10^–4^)	4.9 × 10^–4^	(0.74 × 10^–4^)	0.24 × 10^–4^	(− 0.05 × 10^–4^, 0.54 × 10^–4^)	.156	.245
Splenium (CC)	3.8 × 10^–4^	(0.67 × 10^–4^)	3.7 × 10^–4^	(0.62 × 10^–4^)	0.13 × 10^–4^	(− 0.14 × 10^–4^, 0.41 × 10^–4^)	.392	.544
ORs	4.7 × 10^–4^	(0.56 × 10^–4^)	4.4 × 10^–4^	(0.62 × 10^–4^)	0.29 × 10^–4^	(0.022 × 10^–4^, 0.56 × 10^–4^)	.039	.195
LGNs	7.1 × 10^–4^	(0.37 × 10^–4^)	7.1 × 10^–4^	(0.86 × 10^–4^)	0.031 × 10^–4^	(− 0.28 × 10^–4^, 0.26 × 10^–4^)	.840	.875
IFOFs	4.9 × 10^–4^	(0.42 × 10^–4^)	4.9 × 10^–4^	(0.63 × 10^–4^)	0.077 × 10^–4^	(− 0.15 × 10^–4^, 0.28 × 10^–4^)	.511	.608
V1	7.0 × 10^–4^	(0.37 × 10^–4^)	6.8 × 10^–4^	(0.61 × 10^–5^)	0.17 × 10^–4^	(− 0.049 × 10^–4^, 0.37 × 10^–4^)	.153	.245

The mean difference adjusted for sex and age at 32 years for visual outcomes and sex and age at 26 years for DTI metrics.

*AD* axial diffusivity, *BCVA* best corrected visual acuity, *CC* corpus callosum, *CI* confidence interval, *CS* contrast sensitivity, *FA* fractional anisotropy, *IFOFs* inferior-fronto occipital fasciculus, *LGNs* lateral geniculate nucleus, *MD* mean diffusivity, *ms* milliseconds, *ORs* optic radiations, *p-value*^*adj*^ Benjamin–Hochberg adjusted p-value, *RD* radial diffusivity, *RNFL* retinal nerve fibre layer, *SD* standard deviation, *VLBW* very low birth weight, *V1* primary visual cortex, *µm* micrometre.

^a^Data missing for one VLBW participant and two control participants.

^b^Data missing for six VLBW participants and four control participants.

### DTI metrics in ROIs

The mean difference between the VLBW group and the control group on all DTI metrics in the seven ROIs (genu, body, and splenium of CC, ORs, LGNs, IFOFs, V1) are presented in Table 2. The VLBW group displayed higher AD and higher RD in the genu of CC and higher RD in the ORs than the control group. Adjusted p-values indicated that these mean differences were not statistically significant. The data distribution of DTI metrics for VLBW and control participants are presented in Supplementary Figures B2, B3, and B4 online.

### DTI metrics in ROIs at 26 years as predictors for visual outcomes at 32 years

**Figure 1:**
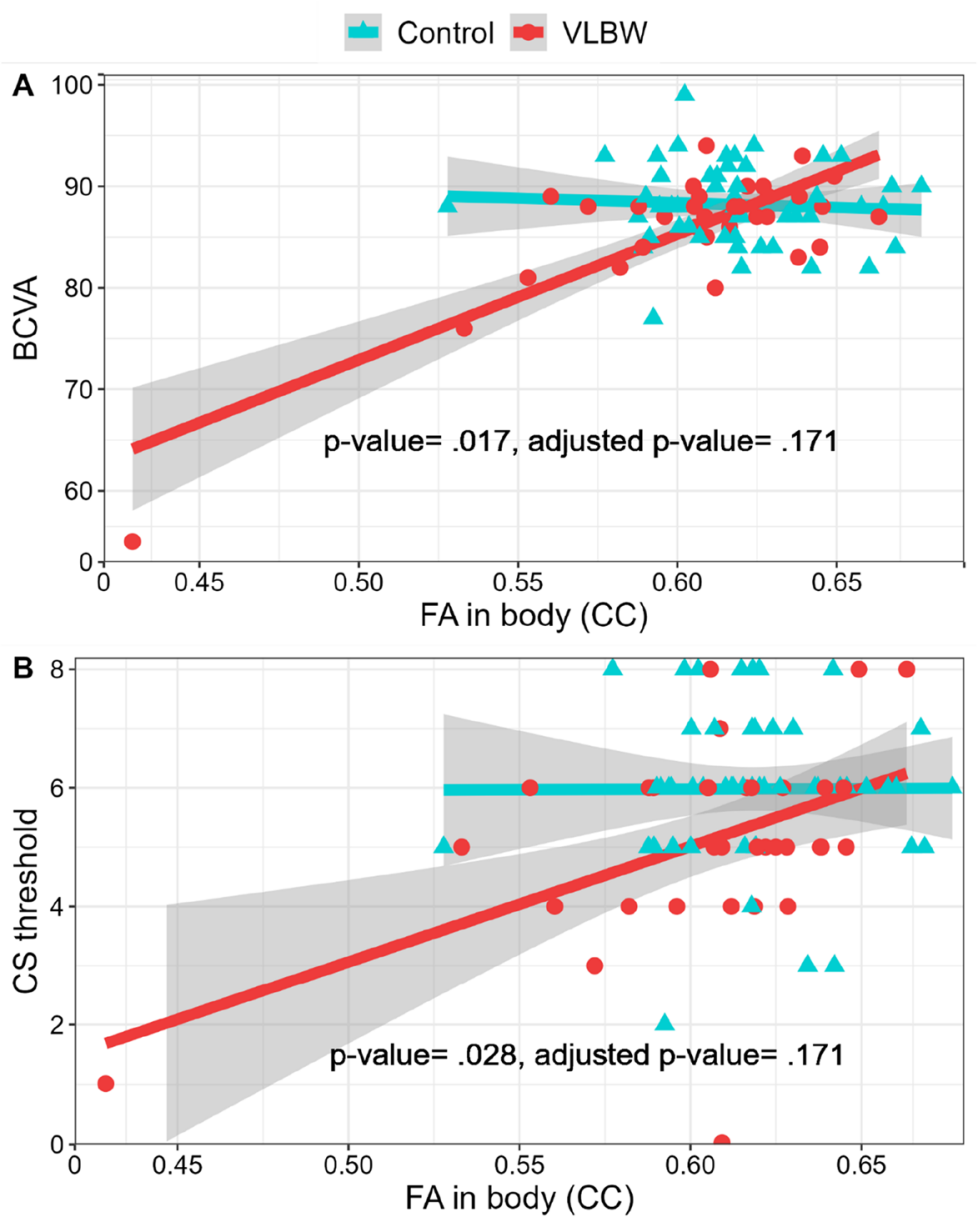
Scatterplots with regression lines, 95% confidence intervals (CI; the grey area surrounding the regression lines), p-value and adjusted p-value for the interaction between FA in body of CC (x-axis, a higher value indicates better white matter integrity) and group (VLBW group in red circles, n = 33, Control group in blue triangles, n = 50) on visual outcomes. A = BCVA (y-axis, 0–100, a higher score represents better visual acuity); B = CS function (y-axis, 0–8, a higher threshold represents better CS). *BCVA *best corrected visual acuity, *CC* corpus callosum, *CS* contrast sensitivity, *FA* fractional anisotropy.

Linear regressions with visual outcomes as dependent variables and between-group interactions between visual outcomes and FA in WM ROIs and MD in GM V1 in the VLBW group and control group are presented in Table 3. The association between BCVA and FA in genu and body of CC and MD in V1 differed between the groups. Within the VLBW group, higher FA in CC, ORs, LGNs, and lower MD in V1 was associated with BCVA. Adjusted p-values indicated that the within-group association between FA in body of CC and visual acuity was statistically significant in the VLBW group (Fig. 1).

**Table 3:** Linear regression with visual outcomes at 32 years as the dependent variable, and group (VLBW versus control) and MD for V1 and FA for each remaining ROI (one at a time) at 26 years and their interaction as covariates, adjusting for age at 32 years and sex.

Visual outcomes	ROIs	VLBW (n = 33)				Controls (n = 50)				FA/MD x group	
		B	(95% CI)	p-value	p-value^adj^	B	(95% CI)	p-value	p-value^adj^	p-value	p-value^adj^
BCVA	FA in genu (CC)	154.5	(− 7.0, 219.4)	.009	.050	− 19.4	(− 60.9, 21.1)	.329	.974	.017	.171
	FA in body (CC)	125.4	(23.0, 156.6)	.000	.000	− 8.3	(− 47.8, 27.4)	.659	.974	.002	.056
	FA in splenium (CC)	96.6	(1.0, 169.3)	.008	.050	10.9	(− 23.5, 57.0)	.532	.974	.058	.171
	FA in ORs	104.5	(20.4, 185.2)	.023	.092	12.5	(− 16.1, 50.5)	.397	.974	.072	.175
	FA in LGNs	248.2	(41.6, 435.7)	.034	.093	3.6	(− 68.7, 80.9)	.924	.998	.059	.171
	FA in IFOFs	106.7	(13.2, 263.7)	.140	.218	1.2	(− 36.2, 45.1)	.951	.998	.136	.254
	MD in V1 (mm^2^/s)	− 10.5 × 10^4^	(− 19.4 × 10^4^, − 1.3 × 10^4^)	.028	.093	0.14 × 10^4^	(− 1.4 × 10^4^, 4.5 × 10^4^)	.788	.974	.025	.171
CS function	FA in genu (CC)	20.5	(− 8.3, 33.9)	.052	.112	− 1.5	(− 15.5, 8.6)	.800	.974	.096	.207
	FA in body (CC)	19.6	(4.4, 30.1)	.001	.014	1.1	(− 12.5, 12.2)	.858	.998	.028	.171
	FA in splenium (CC)	12.3	(− 2.8, 28.7)	.100	.175	2.8	(− 11.5, 12.0)	.619	.974	.345	.483
	FA in ORs	21.0	(5.5, 40.5)	.016	.075	3.6	(− 3.8, 10.5)	.315	.974	.059	.171
	FA in LGNs	35.2	(− 3.3, 67.8)	.058	.116	11.7	(− 10.9, 34.3)	.313	.974	.292	.430
	FA in IFOFs	30.4	(5.4, 63.1)	.035	.093	9.4	(− 5.5, 27.0)	.222	.974	.203	.343
	MD in V1 (mm^2^/s)	− 1.6 × 10^4^	(− 3.2 × 10^4^, 50.4 × 10^4^)	.040	.093	− 0.23 × 10^4^	(− 1.3 × 10^4^, 0.44 × 10^4^)	.199	.974	.075	.175
P100 latency (ms)^a^	FA in genu (CC)	− 15.5	(− 87.5, 100.3)	.734	.917	16.0	(− 53.8, 66.9)	.686	.974	.582	.678
	FA in body (CC)	− 37.9	(− 92.5, 28.7)	.224	.314	15.9	(− 49.5, 58.7)	.640	.974	.222	.345
	FA in splenium (CC)	− 40.4	(− 90.2, 8.4)	.039	.093	36.5	(− 39.8, 74.6)	.254	.974	.041	.171
	FA in ORs	− 72.3	(− 123.9, − 15.8)	.007	.050	− 0.7	(− 62.2, 37.4)	.977	.998	.061	.171
	FA in LGNs	− 20.0	(− 186.2, 136.1)	.808	.917	26.0	(− 75.8, 120.0)	.598	.974	.613	.678
	FA in IFOFs	− 77.1	(− 172.7, 0.0)	.063	.118	9.5	(− 68.9, 51.7)	.793	.974	.110	.220
	MD in V1 (mm^2^/s)	5.3 × 10^4^	(− 1.6 × 10^4^, 12.5 × 10^4^)	.126	.208	− 2.0 × 10^4^	(− 3.9 × 10^4^, 5.6 × 10^4^)	.065	.974	.045	.171
RNFL thickness (µm)^b^	FA in genu (CC)	4.3	(− 28.0, 34.9)	.781	.917	− 7.8	(− 30.2, 14.2)	.431	.974	.518	.631
	FA in body (CC)	2.2	(− 17.8, 28.4)	.874	.917	− 4.7	(− 19.8, 13.8)	.544	.974	.630	.678
	FA in splenium (CC)	6.9	(− 18.3, 31.5)	.587	.783	− 12.9	(− 37.7, 8.9)	.148	.974	.208	.343
	FA in ORs	1.1	(− 22.0, 18.9)	.917	.917	− 9.7	(− 26.7, 6.4)	.170	.974	.394	.501
	FA in LGNs	4.1	(− 46.3, 67.3)	.867	.917	− 0.10	(− 45.4, 47.1)	.998	.998	.900	.933
	FA in IFOFs	14.9	(− 6.04, 39.3)	.172	.253	− 2.3	(− 11.8, 27.8)	.792	.974	.362	.483
	MD in V1 (mm^2^/s)	0.13 × 10^4^	(− 1.8 × 10^4^, 2.4 × 10^4^)	.893	.917	0.19 × 10^4^	(− 1.5 × 10^4^, 0.91 × 10^4^)	.379	.974	.951	.951

*AD* axial diffusivity, *BCVA* best corrected visual acuity, *CC* corpus callosum, *CS* contrast sensitivity, *FA* fractional anisotropy, *IFOFs* inferior-fronto occipital fasciculus, *LGNs* lateral geniculate nucleus, *MD* mean diffusivity, *ms* milliseconds, *ORs* optic radiations, *p-value*^*adj*^ Benjamin–Hochberg adjusted p-value, *RD* radial diffusivity, *RNFL* retinal nerve fibre layer, *ROIs* regions of interest, *SD* standard deviation, *VLBW* very low birth weight, *V1* primary visual cortex, *µm* micrometre.

^a^Data missing for one VLBW participant and two control participants.

^b^Data missing for six VLBW participants and four control participants.

For CS function, the association with FA in the body of CC differed between the groups and higher FA in the body of CC, ORs, and IFOFs and MD in V1 was associated with better CS function within the VLBW group. Adjusted p-values indicated that the within-group association between FA in body of CC and contrast sensitivity was statistically significant in the VLBW group (Fig. 1).

For the visual pathway function (P100 latency), the association with FA in the splenium of CC and MD in V1 differed between the groups. Within the VLBW group, higher FA in the splenium of CC and ORs was associated with a P100 latency closer to 100 ms. Adjusted p-values indicated that there were no significant between-group or within-group associations between DTI metrics and P100 latency.

There were no differences between or within the groups for the association between central RNFL thickness and FA or MD in any ROIs.

The association of RD in CC, LGNs, and V1 with BCVA; RD in the splenium of CC and V1 with P100 latency; and RD in the LGNs with central RNFL thickness differed between the groups. Adjusted p-values indicated that the association between lower RD in the body of CC and BCVA within the VLBW remained significant. Within the VLBW group, lower RD generally indicated better BCVA and CS function and central thicker RNFL in LGNs (Supplementary Table B1 online). The association between AD in V1 with BCVA differed between the groups. Within the control group, higher AD in LGNs and IFOFs was significantly associated with a P100 latency closer to 100 ms (Supplementary Table B2 online).

#### Sensitivity analysis

A sensitivity analysis excluding participants with NSI (VLBW n = 4) were performed for all primary linear regression analyses. Two of the participants with NSI had extreme scores (± 2SD) in the DTI metrics (Supplementary Figures B2, B3 and B4 online). After the sensitivity analysis were performed, the VLBW group still showed lower AD in genu than the control group. Also, the VLBW participants still had a lower CS function of almost one threshold of contrast level and a thicker RNFL than controls (Supplementary Table C1 online). Adjusted p-values showed no statistically significant mean differences between the groups. Within the VLBW group, higher FA in LGNs was still associated with better BCVA, and higher FA in the body of CC and ORs was associated with better CS function (Supplementary Table C2 online), but after p-value adjustment there were no statistically significant findings.

## Discussion

In this study, we found that the VLBW group on average had lower CS function and a thicker central RNFL at 32 years, higher AD, and higher RD in the genu of CC, and higher RD in the OR at 26 years compared to the control group. In addition, VLBW participants displayed a slightly longer P100 latency than controls. Moreover, the results indicated that lower FA in several ROIs and higher MD in V1 were associated with reduced visual function in adults born with VLBW, with the strongest associations in the CC. Sensitivity analyses excluding individuals with NSI no longer showed any significant associations after adjusting p-values, but estimated mean differences and associations indicated the same tendency of reduced visual function with lower FA within the VLBW group.

A strength of this study is the longitudinal cohort design, with participants included at birth and assessed at several time points. Also, the brain MRI scans, and processing of the DTI data were completed by a researcher blinded for group status. The ophthalmologist performing the visual assessment and the neurophysiologist performing the PR-VEP examination at 32 years were also blinded, reducing the risk of information bias. In a longitudinal study, there is always a challenge with loss to follow-up, as was the case for this study. Some loss to follow-up may be due to the 32-year assessment partly being carried out during the covid-19 pandemic. There were some, but only small, differences in background characteristics between participants and non-participants. However, the slightly lower gestational age, birth weight, Apgar scores after 1 min, and maternal age among non-participants would if anything result in an underestimation of differences between the VLBW and control group.

In cohort studies, the potential of confounders and covariates affecting the results are difficult to avoid. We therefore chose to adjust the analyses for the covariates age and sex. We do recognise that parental SES could be a potential confounder affecting visual outcomes and DTI metrics. Unfortunately, several of the participants at 26 and 32 years were missing data on parental SES from the 14- and 19-years assessment. Since we performed complete cases analyses in this study several of these participants would have been excluded from the analysis by including parental SES as a covariate, consequently affecting the results. Moreover, a recent review of all published papers in the NTNU LBW Life study that includes neuroimaging data showed that parental SES was not associated with most of the health outcomes in the cohort^1^.

To explore altered brain microstructure, we used DTI metrics. DTI can be used as a proxy for characterizing white and grey matter microstructure. The regions of interest that were used to extract DTI metrics in this study was carefully checked for correct placement by going through all slices for each participant and visual quality control was performed by an experienced MRI analyst (LE). However, DTI has some methodological limitations that should be mentioned. In addition to being vulnerable for motion artefacts^20^, its assumptions are often violated because the change in DTI metrics is related to various physiological processes which can affect the direction and strength of the DTI data^21^. Furthermore, due to the partial volume effects between adjacent white matter tracts, voxels containing multiple fibre orientations are frequent, and in these cases, the diffusion tensor can be unreliable^22^. High-angular resolution diffusion imaging is a more robust sequence that makes the extraction of fibre orientations and interpreting DTI metrics more reliable^23^. However, this sequence requires a longer scan time which have caused data loss due to movement artefacts in the earlier DTI assessments of participants in the NTNU LBW Life study. The HARDI sequences was, therefore, not used to acquire DTI data in this study.

The relatively small sample size limits the generalizability of the study. Findings of no significant differences should be interpreted cautiously since the confidence intervals may include smaller effects. Due to the small sample size, the potential correlation between the DTI metrics, and the explorative nature of this study, the results are shown with unadjusted, and Benjamin-Hochberg adjusted p-values. A p-value adjustment can reduce the chance of Type I error at the expense of increasing the likelihood of Type II errors^24^. To reduce the number of tests, we have only included linear regression analyses of the association between FA/MD and visual outcomes and the mean difference between the groups on these outcomes as primary results. Analyses including AD and RD in ROIs as predictors, are supplementary results to support the discussion of the main findings from the primary analyses. However, we recognize that multiple testing can affect the statistical power of the study. Consequently, adjusted p-values calculated with the Benjamin-Hochberg approach is presented in all tables^25^.

We chose a priori to include all participants with available data in the primary analyses to get a representative impression of all VLBW individuals in this cohort. However, the VLBW group included four participants with NSI that most likely affected the visual outcomes and DTI metrics to some degree. These participants were hence excluded in sensitivity analyses. Two of the four participants with NSI displayed a deviation from the mean of ± 2 SD in some DTI metrics, which could explain the discrepancy in results between the main analyses and the sensitivity analyses. Indeed, previous studies have indicated that cerebral palsy is a risk factor for later visual problems in individuals born preterm^26^ and that children and adolescents born very preterm display an atypical development of callosal maturation which is associated with cognitive dysfunction and reduced IQ^27^. It, therefore, seems conceivable that the largest discrepancies in results could be explained by the impact that NSI have on visual outcomes and DTI metrics in preterm populations.

Lower CS function in the VLBW group has previously been shown in our cohort at 14 years^3^ using a slightly different method with non-standardized lighting conditions. Since, we found similar results in adulthood with the use of gold standard contrast sensitivity assessment in standardized light conditions, the similar results indicates that reduced CS function may persist from adolescence into adulthood. Moreover, the results align with findings of reduced CS function in children at age 10 born preterm with VLBW (Larsson, Rydberg et al. 2006), supporting the notion that reduced CS function in those born VLBW is present across these ages. The VLBW group showed slightly longer P100 latencies than the control group. This has previously been demonstrated in 4–6 year old children born preterm with VLBW^7^, but as far as we know, this is the first study showing similar findings in adults born preterm with VLBW.

In our sample, BCVA did not differ between the groups. An explanation might be that the participants in the VLBW group at 32 years had good visual acuity with a mean BCVA ETDRS letter score of almost 85, which is regarded as normal vision clinically. The visual function findings in this study might indicate that the CS test with low spatial frequencies is a more sensitive measure of day-to-day vision than the BCVA letter score, which tests only consists of high contrast stimuli. Therefore, to assess the visual function in more detail, CS function should be evaluated when visual acuity is within a normal range.

The central RNFL thickness differed between the groups with thicker central RNFL (A1 of the ETDRS grid) in the VLBW group compared with controls. Our data are in line with the few existing studies investigating RNFL, such as the EPICure study that reported a thicker RNFL in A1 of the EDTRS grid in a group of 19-year-olds born extremely preterm compared to controls^6^. The results also coincide with findings of thicker central retinal layers with earlier gestational age in infants born extremely preterm^28^, 5 to -16-year-old children born preterm^29^. The centrifugal movement of inner retinal layers to the periphery and migration of outer photoreceptor layers towards the centre of the retina^30^ occurs during late gestation and preterm birth therefore disrupt these processes^28^ which can result in a thicker RNFL in the inner layers, as shown in this study.

We did not find the WM microstructure pattern of lower FA and higher MD along the visual pathway in adults born preterm with VLBW. However, RD in the genu of CC and ORs and AD in the genu of CC were higher in the VLBW group compared with the control group at 26 years. These findings coincides with a study of 27–29 years old VLBW adults in New Zealand that showed higher AD and RD in CC compared with controls^17^.

Findings from the association analyses indicated that increased FA in the CC was associated with better visual acuity (genu, body, and splenium of CC), CS function (body of CC) and a P100 latency closer to 100 ms (splenium of CC), within the VLBW group. These results extend previous findings in this cohort in that visual acuity was positively correlated to FA in the splenium and body of CC in the same cohort at adolescence^19^. Moreover, a previous study from Poland has reported an association between abnormal stereoscopic vision and lower FA in the genu and body of CC in 4-year old children born with VLBW^31^.

The observed higher AD in the genu of CC in the VLBW group compared with the control group might suggest that poorer WM microstructure is not caused by axonal injury or poor fiber organization within the voxels (which would be indicated by decreased AD). Rather, hypo- or demyelination and low axon density may be a source^17^, as indicated by the higher RD in the genu of CC and ORs in the VLBW group compared with the control group. Taken together, the results suggested that lower RD (indicating normal myelination) in the body of CC was associated with better BCVA and CS function, while lower RD in LGNs and V1 also was associated with better BCVA within the VLBW group. The findings underline the importance of interpreting AD and RD metrics when interpretating FA findings, and higher RD in this preterm population can be explained by the disrupting effect that preterm birth has on normal development of myelin-producing cells, causing abnormal white matter development^32,33^.

Several studies have identified the CC as an important site of altered WM in studies involving preterm born individuals^34,35^. This is also supported by our findings of an association between FA in genu and body of CC with visual acuity, between FA in the body of CC and CS function, and FA in the splenium of CC and P100 latency. The importance of CC for visual information flow between the hemispheres is well-established as it is the largest WM structure, linking the cortical areas that represent opposite visual hemifields^5^. However, evidence connecting CC is to visual outcomes are scarce. The parallel visual processing model suggests that visual processing is more efficient across hemifields than within a single hemifield, which might explain the importance of a CC packed with myelinated axons for efficient transmission of visual information between the hemispheres and, thereby, optimal visual function^31,36^.

The participants were born in the 1980s, and one may question whether the findings in this study apply to those born preterm today. However, cohort studies in children born preterm in the 2000s^31,37^ report similar outcomes with regard to WM microstructure and visual outcomes. The modern era of neonatal care has led to enhanced survival rates. Still, even though some studies show that this improvement could be associated with a reduction in the severity of WM injury among preterm survivors^38^, it has been accompanied by an increase in the number of preterm survivors with long-term neurodevelopmental difficulties^39^.

Our findings indicate that the reduced CS function previously found in VLBW adolescents compared with term-born controls in the NTNU LBW Life study^3^ persists into adulthood. Since reduced vision among preterm born individuals is not always caused by ophthalmological sequela^8^, the early detection of visual dysfunction caused by central mechanisms by the use of MRI scans, PR-VEPs and/or OCT could be applied to improve the follow-up treatment in the absence of any ophthalmological disease that could otherwise explain the reduced vision that is observed in this clinical population.

## Conclusion

The findings from this study showed that CS function was poorer, central retinal nerve fiber layer was thicker, axial and radial diffusivity in the genu of the CC was higher, and radial diffusivity in optic radiations was higher in the VLBW group compared with the term-born control group. Moreover, the findings suggest that DTI metrics in white matter tracts along the visual pathway, especially in CC, could help understand the etiology of poorer visual outcomes in individuals born preterm. Diffusion tensor imaging as a tool for exploring clinical markers for visual outcomes could help foster tailored strategies for individuals born preterm and should be further explored in future studies with larger populations of adults born preterm.

## Material and methods

### Study design

This study is part of an extensive longitudinal cohort study of a group of preterm-born individuals with VLBW (birth weight ≤ 1500 g) and a term-born control group (gestational age ≥ 37 weeks with a birth weight ≥ 10th percentile). Participants in the VLBW group were born in 1986–1988 and admitted to the Neonatal Intensive Care Unit (NICU) at St. Olavs Hospital, Trondheim University Hospital, in Norway. Participants in the control group were born to mothers recruited from the Trondheim area in pregnancy during the same years. The participants were invited to follow-up assessments at several time points from childhood to adulthood^1^. In this study, we used data from a brain MRI assessment at 26 years of age and a visual assessment at 32 years of age, the latter carried out between 2019 and 2021.

### VLBW group

**Figure 2:**
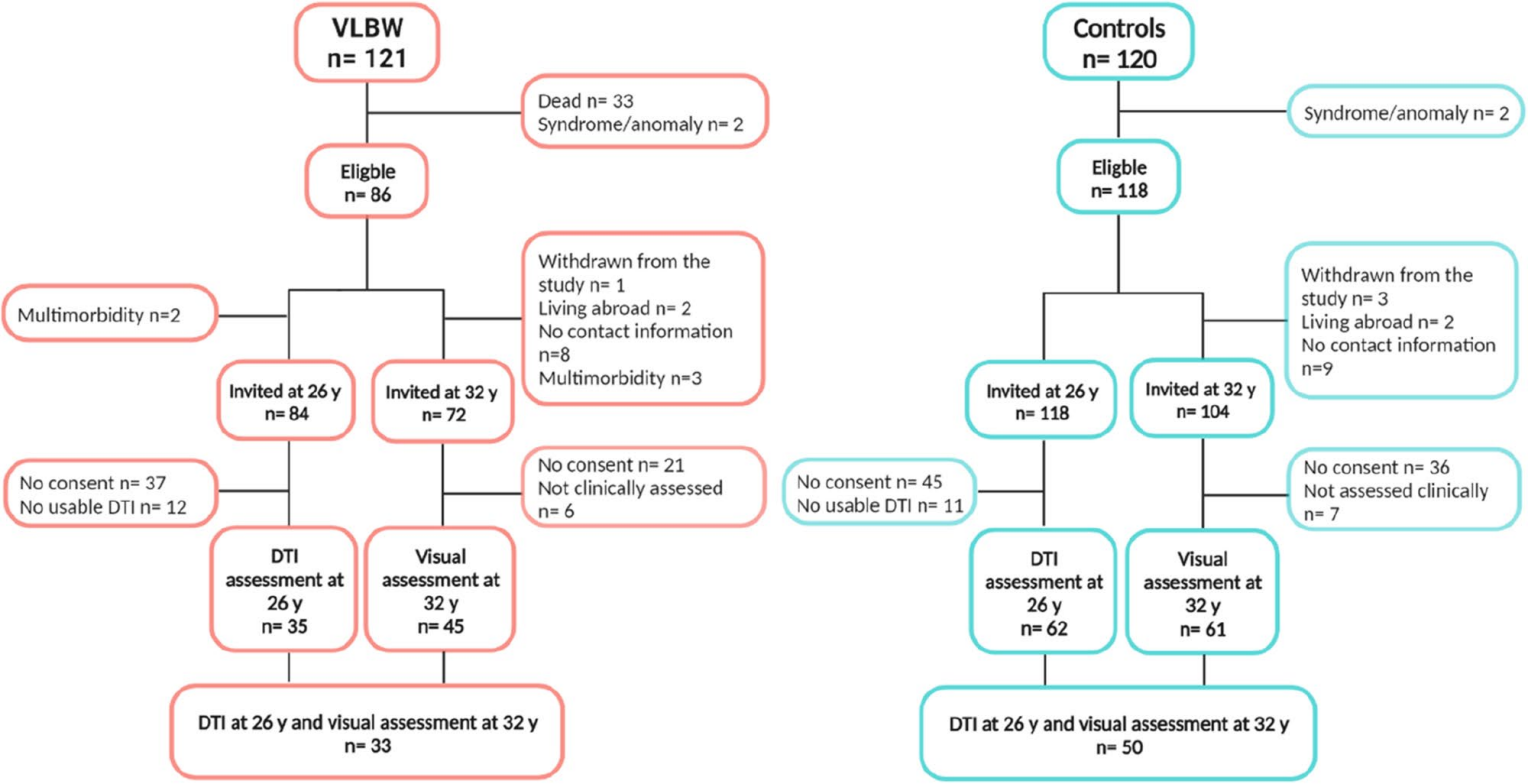
The flow of participants at 32 years and 26 years of age. *DTI *diffusion tensor imaging, *VLBW *very low birth weight.

Figure 2 illustrates the flow of participants in the study. The VLBW group originally consisted of 121 individuals with a birth weight ≤ 1500 g. Of these, 33 died in the neonatal period and two were excluded due to congenital syndrome/anomaly. Of the 86 eligible participants, two were excluded due to multimorbidity at the 26 years assessment, leaving 84 VLBW individuals that were invited for the brain MRI assessment at 26 years. Of these, 37 did not consent to participation, and 12 had no usable DTI assessment due to movement artefacts, leaving 35 participants with DTI data. Prior to the 32 years assessment, one participant had withdrawn from the study, eight were without contact information and two were living abroad. Also, three participants were excluded due to multimorbidity, leaving 72 controls that were invited for the clinical visual assessment at 32 years. Of these, 21 did not consent and six only answered questionnaires and were not assessed clinically because they were unable to attend the clinical assessment. Finally, 45 completed the clinical visual assessment at 32 years. In all, 33 VLBW individuals completed assessment of visual outcomes at 32 years and DTI assessment at 26 years and were included in the analyses.

### Term-born control group

The control group originally consisted of 120 individuals with birth weight ≥ 10th percentile for gestational age, corrected for sex and parity^40^. Of these, two were excluded due to congenital syndrome/anomaly, leaving 118 controls that were eligible and invited for the brain MRI at 26 years. Of these, 45 did not consent to participation, and 11 had no usable DTI due to movement artefacts, leaving 62 participants with DTI data. Prior to the 32 years assessment, three participants had withdrawn from the study, nine were without contact information, and two were living abroad. Therefore, 104 controls were invited at 32 years. Of these, 36 did not consent to participation, and seven only answered questionnaires and were not assessed clinically because they were unable to attend the clinical assessment. Finally, 61 completed the clinical visual assessment at 32 years. In all, 50 term-born controls completed assessment of visual outcomes at 32 years and DTI assessment at 26 years and were included in the analyses (Fig. 2).

### Non-participants

Non-participants included those who did not consent to participation and were not assessed clinically at 32 years because they did not meet for the clinical assessment or did not have a usable DTI at 26 years. Descriptive statistics are presented in Supplementary Table A1 online. In the VLBW group, non-participants had slightly lower gestational age, birth weight, Apgar score after 1 min, and maternal age at delivery than participants. In addition, background information indicated that there were fewer males among participants than among non-participants. In the control group, non-participants had slightly lower gestational age and maternal age at delivery than participants.

### Clinical characteristics

Perinatal background data included maternal age, parental socioeconomic status (SES), birth weight, gestational age, head circumference at birth, Apgar scores, age at assessments, sex, intraventricular haemorrhage (IVH, grade 1–4) and/or, periventricular leukomalacia, and neurosensory impairment (NSI). The presence of NSI was defined as blindness, use of a hearing aid, cerebral palsy diagnosed by a paediatrician at the 14-year follow-up or self-reported at the 32-year follow-up, and/or IQ score below 70 assessed by a psychologist with the Wechsler Adult Intelligence Scale-Third edition at 19 years. If data on IQ was not available from 19 years, we used data on estimated IQ based on two subtests of the Wechsler Intelligence Scale for Children-Third edition at 14 years, and if that was not available, we used data on IQ measured with the Wechsler Preschool and Primary Scale of Intelligence-Revised at 5 years. Parental SES was calculated according to Hollingshead’s Two Factor Index of Social Position, based on information on parent’s education and occupation^41^. The score ranges from 1 (low) to 5 (high). Parental SES was collected at the 14 years assessment and supplemented at the 19 years assessment.

### Visual outcomes

The participants underwent a clinical visual assessment of visual function and visual structure. An ophthalmologist (APJ) performed all tests on both eyes separately and bilaterally, starting with the right eye. The participants included in the NTNU LBW Life study were born in the 1980s before Norway implemented systematic ROP screening. Therefore, we do not have detailed information regarding the ROP status of the participants. However, our project ophthalmologist (APJ) thoroughly checked the participants’ medical journals, and a clinical visual examination of all participants showed no sign of any previous ROP diagnoses that would affect the visual outcomes in this paper. All tests for visual outcomes are illustrated in Supplementary Figure A online.

#### Visual function

After subjective refraction was performed, the best-corrected visual acuity (BCVA) letter score was measured with the Early Treatment Diabetic Retinopathy Study (ETDRS) chart using a standardized protocol at 4 m^42^ under standardized light conditions. The better eye was defined as the eye with the highest BCVA letter score and used in all analyses. If the BCVA letter score was equal, the right eye was chosen as the better eye. The better eye was used for all analyses because the better eye will be the preferred eye and, therefore, a more accurate measure of visual function. Moreover, using both eyes may distort the BCVA letter score due to potential non-central nervous system injury (such as amblyopia or strabismus), affecting the BCVA letter score in the worse eye^4^.

Contrast sensitivity (CS) was tested with the best-refracted correction at a constant mean luminance of 85 cd/m^2^ (no glare). We used the CSV 1000E chart (Vector Vision, Haag-Streit, Harlow, UK) consisting of four rows and eight columns of sine-wave gratings, where participants were asked to identify the grating pattern in each column. In eight contrast levels, participants were tested at four spatial frequencies with four cycles per degree (cpd; 3, 6, 12, and 18 cycles per degree). The lowest level of contrast the participants could see in each spatial frequency was noted. Cycles per degree measures how many lines you can distinguish in a degree of the visual field. Only 6 cpd was included in this study because low spatial frequencies, and especially 6 cpd, are most sensitive to detecting objects at low contrasts and have the best predictive value at the peak of the contrast sensitivity curve^4,43–45^.

#### Retinal structure

Optical coherence tomography (OCT) images were obtained using a Heidelberg Spectralis machine (Heidelberg, Germany). The ETDRS grid was used to extract the central retinal nerve fibre layer (RNFL) thickness. The circular ETDRS grid is divided into nine macular sectors defined by three rings of 1 mm, 3 mm, or 6 mm in diameter, used to anatomically divide the retina to measure the thickness of different retinal layers within the sectors^46^. The mean RNFL thickness (µm) was measured in the innermost ring of the retina, corresponding to the A1 area of the ETDRS grid, measuring 1 mm in diameter.

#### Visual pathway function

Pattern-reversal visual evoked potentials (PR-VEPs) from the left and right eye were recorded on a Dantec Keypoint G4 workstation (Natus Medical, USA) using a View Sonic Graphics Series G70fmb CRT monitor as the pattern generator. Electrodes were placed according to the 10–20 system on occipital, frontal, and parietal areas (Oz, Fz, and Pz)^47^. PR-VEPs were recorded from the parietal (Pz) and occipital (Oz) midline and referred to the mid-frontal electrode (Fz), according to the ISCEV standards^48^. Impedance was < 5 kΩ, and a 1 Hz–1 kHz filter was used.

The participants were seated in a relaxed position in a chair with neck support. The eye that was not tested was covered with an eyepatch. The PR-VEP task consisted of high-contrast black-and-white checks with a red fixation point in the middle of the checkboard. The PR-VEP recording was performed in a dark room at 1 m from the CTR monitor. PR-VEPs were recorded in one eye at a time with a 33' (24 × 32) check size with 160 stimulations per run and a stimulations frequency of 1 reversal per second (rps). At least two runs, three runs for most participants, for each eye were conducted to achieve a relative noise-free averaged VEP and to assess the reproducibility of the response runs. Two repeatable response runs were required as a minimum. Repeatability was generally excellent as visually evaluated by the specialist. Two or three repeatable response runs were then averaged in the software to get the final averaged VEP curve for the measurements. An experienced clinical neurophysiology (AG) specialist visually identified and placed cursors on N70, P100, and N145 peaks. The P100 latency (ms) was measured from stimulus onset to the peak of the P100 wave and was used in the analysis because of its clinical relevance due to its high amplitude, less variability between participants, and minimal interocular asymmetry^49^.

### Brain MRI

#### Image acquisition

At 26 years, DTI from participants was acquired on a 3 T Siemens Skyra equipped with a 32-channel head matrix coil (Siemens AG, Erlangen, Germany). To reduce noise from movement, foam pads were placed around the participants' heads. The DTI was acquired with a single-shot balanced echo EPI sequence with b = 1000 s/mm^2^ and b = 2000s/mm^2^ in 30 non-collinear directions using the following parameters: TR = 8800 ms, TE = 95 ms, FOV = 240 × 240 mm, slice thickness 2.5 mm, acquisition matrix 96 × 96, giving isotropic voxels of 2.5 mm. Full brain coverage was obtained with 60 axial slices without a gap. Five images without diffusion weighting (b0) were acquired for each slice. Sixty images with diffusion gradients were also acquired for each slice (consisting of 30 images with b = 1000 s/mm^2^ and 30 with b = 2000s/mm^2^). To correct the image distortion caused by magnetic susceptibility artefacts, two additional b0 images were acquired with opposite phase-encode polarity.

#### Image analysis

The DTI analyses were performed with the tools of the FMRIB Software Library (FSL; Oxford Centre for Functional MRI of the Brain, UK; www.fmrib.ox.ac.uk/fsl). Image artefacts due to motion and eddy current distortions were minimized by registration of all DTI acquisitions to the mean b = 0 image using affine registration. Image distortion caused by magnetic susceptibility artefacts was minimized with a nonlinear B0-unwarping method using paired images with opposite phase-encode polarities (Holland et al., 2010). The brain was extracted using the Brain Extraction Tool (BET, part of FSL). FMRIB's Diffusion Toolbox (FDT) was used to fit a diffusion tensor model to the raw diffusion data in each voxel. Voxel-wise maps of FA, MD, axial (AD; λ1), and radial diffusivity (RD; (λ2 + λ3)/2) were calculated for the VLBW and control groups. Visual quality control of the data was performed by an experienced analyst (LE).

#### White matter tract regions and DTI metrics of interest

**Figure 3:**
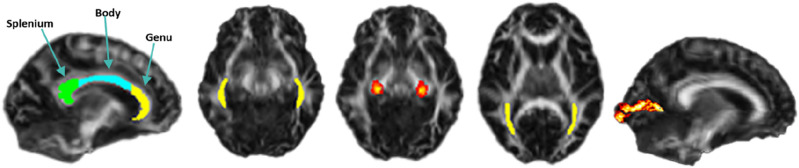
Cortical grey matter and white matter tract regions of interest along the visual pathway. From the left: corpus callosum (including the splenium, body, and genu), optic radiations, lateral geniculate nucleus, inferior-fronto occipital fasciculus, and primary visual cortex.

Regions of interest (ROIs) in white matter tracts and primary visual cortex were chosen based on which brain regions are involved in the visual pathway and visual processing, and these were: corpus callosum (body, genu, splenium; CC), lateral geniculate nucleus (LGNs), optic radiations (ORs), inferior fronto-occipital fasciculus (IFOFs), the primary visual cortex (V1) in the right and left hemispheres^19,50,51^. They are presented in Fig. 3. The ROIs were manually created based on masks from the John Hopkins University (JHU) white-matter labels atlas^52^ and the Jülich histological atlas in FSL^53,54^. The masks were overlayed on the mean skeleton FA from TBSS and checked for each participant to confirm that the ROIs were correctly placed.

### Statistical analysis

Descriptive statistics are presented as mean with standard deviation for scale variables and counts with percentages for categorical variables. We first used linear regression with visual outcomes and DTI metrics for each ROI, one at a time, as dependent variables, with group (VLBW versus control) as the independent variable and age and sex as covariates. For the association analyses, we used linear regression with one visual outcome measure at a time as the dependent variable. Age and sex, FA for the ROIs in the corpus callosum, optic radiations, lateral geniculate nucleus, inferior-fronto occipital fasciculus and MD for V1 (one at a time), group and the interaction between group and FA/MD in ROIs were entered as independent variables. This analysis was repeated with AD and RD as DTI metrics.

The normality of residuals was assessed by visual inspection of Q-Q plots. Since some deviations from normality were found, we used bootstrapping (2000 bootstrap samples and the bias-corrected and accelerated (BCa) method) in all regression analyses. We also performed a sensitivity analysis excluding the four participants with NSI. We report 95% confidence intervals where relevant and regard two-sided p-values < 0.05 to represent statistical significance. In addition, we adjusted for multiple compariasons and report Benjamin-Hochberg adjusted p-values for all statistical analyses. Statistical analyses were performed using SPSS 28.0 (IBM SPSS Statistics, NY, USA) and RStudio 4.1.2 (PBC, Boston, MA, USA).

### Ethics

The study was conducted at the Department of Ophthalmology, Trondheim University Hospital in Norway following the guidelines of the Declaration of Helsinki and was approved by the Regional Committee for Medical Research Ethics in Central Norway (23879). Written informed consent was obtained from all participants.

### Supplementary Information

Supplementary Information.

## Data Availability

The datasets generated and/or analysed during the current study are not publicly available because permission has not been applied for from neither the participants nor the Ethical Committee. The R and SPSS scripts/syntax are available from the corresponding author on reasonable request.
