# Supplementary material for: Visual outcomes and their association with grey and white matter microstructure in adults born preterm with very low birth weight
Source: Sci Rep. 2024 Feb 1;14:2624. doi: 10.1038/s41598-024-52836-4 (PMC10831077; doi:10.1038/s41598-024-52836-4)
Supplement: Supplementary file 1 — Supplementary Information. [file 41598_2024_52836_MOESM1_ESM.pdf]

Visual outcomes and their association with grey and white matter microstructure in adults born preterm with very low birth weight

**Sigrid Hegna Ingvaldsen[1, 2], Anna Perregaard Jørgensen[1], Arnstein Grøtting[3], Trond Sand[1,3,4], Live Eikenes[5], Asta K. Håberg[1, 5], Marit S. Indredavik[6], Stian Lydersen[7], Dordi Austeng[1, 2], Tora Sund Morken[1, 2], Kari Anne I. Evensen[6, 8]*

[1] Department of Neuromedicine and Movement Science, NTNU Norwegian University of Science and Technology, Trondheim, Norway

[2] Department of Ophthalmology, St. Olav Hospital, Trondheim University Hospital, Trondheim, Norway

[3] Department of Neurology and Clinical Neurophysiology, St. Olavs Hospital, Trondheim University Hospital, Trondheim, Norway

[4] NorHEAD - Norwegian Centre for Headache Research, Department of Neuromedicine and Movement Science, NTNU Norwegian University of Science and Technology Trondheim, Norway

[5] Department of Radiology and Nuclear Medicine, MR-center, Trondheim University Hospital, Trondheim, Norway

[6] Department of Clinical and Molecular Medicine, Norwegian University of Science and Technology, Trondheim, Norway

[7] Regional Centre for Child and Youth Mental Health and Child Welfare, Department of Mental Health, Faculty of Medicine and Health Sciences, Norwegian University of Science and Technology, Trondheim, Norway

[8] Department of Physiotherapy, Oslo Metropolitan University, Oslo, Norway

Corresponding author:

Sigrid Hegna Ingvaldsen

Email: sigrid.h.ingvaldsen@ntnu.no

Phone: +47 915 13 022

Supplemental Table A1 Mean (SD) of background characteristics in participants and non-participants

	VLBW				Controls			
	Participants (n= 33)		Non-participants (n=39)		Participants (n= 50)		Non-participants (n= 54)	
	**Mean**	**(SD)**	**Mean**	**(SD)**	**Mean**	**(SD)**	**Mean**	**(SD)**
Maternal age (years)[a]	30.0	(5.3)	26.3	(3.6)	31.3	(4.5)	29.2	(3.7)
Parental SES (1-5)[b]	3.3	(1.6)	3.3	(1.2)	3.9	(1.1)	3.6	(1.1)
Birth weight (g)	1274.7	(203.6)	1091.3	(228.8)	3736.1	(472.4)	3663.0	(423.7)
Gestational age (weeks)	29.6	(2.8)	28.2	(2.5)	40.0	(1.2)	39.5	(1.2)
Head circumference at birth (cm)[c]	27.5	(2.2)	26.5	(2.4)	35.6	(1.1)	35.3	(1.2)
Apgar score after 1 min[d]	7.1	(1.7)	6.0	(2.8)	8.9	(0.4)	8.9	(0.3)
Apgar score after 5 min[e]	8.8	(1.1)	8.2	(2.0)	9.9	(0.4)	9.7	(1.3)
Age at Brain MRI (26 y)[f]	26.2	(0.7)	26.4	(0.5)	26.5	(0.4)	26.5	(0.5)
Age at clinical assessment (32 y)[g]	32.4	(0.8)	32.6	(0.7)	32.5	(0.5)	32.9	(0.4)
	**n**	**(%)**	**n**	**(%)**	**n**	**(%)**	**n**	**(%)**
Males	13	(39.4)	27	(69.2)	21	(42.0)	27	(50.0)
Intraventricular haemorrhage (grade 1-4) and/or periventricular leukomalacia[h]	3	(9.1)	4	(10.3)	0	NA	0	NA
Neurosensory impairments (yes)[i]	3	(9.1)	6	(19.8)	0	NA	1	(2.0)

MRI= magnetic resonance imaging; NA= not applicable; SD= standard deviation; SES= socioeconomic status (1-5, where five is highest); VLBW= very low birth weight.

[a] Data missing for one VLBW non-participant, one control participant and three control non-participants [b] Data missing for two VLBW participants, eight VLBW non-participants, eight control participants and seventeen control non-participants [c] Data missing for five VLBW participants, 11 VLBW non-participants, three control participants and two control non-participants [d] Data missing for one VLBW participant, one VLBW non-participant, three control participants and three control non-participants [e] Data missing for one VLBW participant, two VLBW non-participants, three control participants and two control non-participants [f] Data missing for seventeen VLBW non-participants and twenty-four control non-participants [g] Data missing for twenty-seven VLBW non-participants, thirty-six control non-participants [h] Data missing for one VLBW participant eight VLBW non-participants [i] Data missing for seven VLBW non-participants, three control participants and 3 control con-participants

Supplemental Table B1. Linear regression with visual outcomes at 32 years as the dependent variable, and group (VLBW versus control) and RD for ROIs (one at a time) at 26 years and their interaction as covariates, adjusting for age at 32 years and sex

		VLBW (n= 33)				Controls (n= 50)				RD x group	
**Visual outcomes**	**RD in ROIs** (mm^2/s)	**B**	**(95% CI)**	**p-value**	**p-value[adj]**	**B**	**(95% CI)**	**p-value**	**p-value[adj]**	**p-value**	**p-value[adj]**
**BCVA**	Genu (CC)	-6.6·10^4	(-11.1·10^4, 0.28·10^4)	.006	.056	-0.12·10^4	(-2.9·10^4, 1.3·10^4)	.881	.901	.011	.106
	Body (CC)	-7.9·10^4	(-10.7·10^4, -.60·10^4)	.000	.000	-0.01·10^4	(-2.8·10^4, 1.2·10^4)	.858	.901	.002	.056
	Splenium (CC)	-6.9·10^4	(-11.3·10^4, 0.49·10^4)	.008	.056	-0.24·10^4	(-3.6·10^4, 1.2·10^4)	.692	.807	.018	.106
	ORs	-5.8·10^4	(-11.8·10^4, -0.19·10^4)	.037	.158	-0.40·10^4	(-3.1·10^4, 0.99·10^4)	.588	.778	.058	.152
	LGNs	-8.8·10^4	(-18.5·10^4, -0.60·10^4)	.045	.158	0.26·10^4	(-2.3·10^4, 3.9·10^4)	.488	.727	.027	.108
	IFOFs	-4.6·10^4	(-12.6·10^4, 1.9·10^4)	.207	.414	0.12·10^4	(-2.8·10^4, 3.8·10^4)	.801	.897	.154	.240
	V1	-11.4·10^4	(-19.9·10^4, -1.4·10^4)	.015	.084	0.23·10^4	(-3.4·10^4, 5.1·10^4)	.663	.807	.012	.106
**CS function**	Genu (CC)	-0.73·10^4	(-1.5·10^4, 0.55·10^4)	.135	.315	-0.31·10^4	(-0.89·10^4, 0.19·10^4)	.169	.430	.447	.541
	Body (CC)	-1.1·10^4	(-1.7·10^4, -0.02·10^4)	.004	.056	-0.33·10^4	(-1.2·10^4, -0.11·10^4)	.127	.409	.108	.201
	Splenium (CC)	-0.88·10^4	(-1.7·10^4, 0.30·10^4)	.062	.095	-0.24·10^4	(-0.92·10^4, 0.23·10^4)	.120	.347	.211	.311
	ORs	-1.2·10^4	(-2.3·10^4, -0.30·10^4)	.026	.095	-0.18·10^4	(-0.75·10^4, -0.52·10^4)	.297	.554	.065	.152
	LGNs	-0.53·10^4	(-2.5·10^4, 1.36·10^4)	.556	.649	-0.14·10^4	(-1.0·10^4, 0.76·10^4)	.280	.554	.658	.709
	IFOFs	-1.6·10^4	(-3.3·10^4, -0.27·10^4)	.027	.095	-0.17·10^4	(-1.4·10^4, 1.2·10^4)	.341	.562	.057	.152
	V1	-1.7·10^4	(-3.3·10^4, -0.01·10^4)	.031	.096	-0.24·10^4	(-1.3·10^4, 0.39·10^4)	.188	.439	.060	.152
**P100 latency (ms)[a]**	Genu (CC)	1.9·10^4	(-1.5·10^4, 6.6·10^4)	.170	.280	-0.22·10^4	(-2.4·10^4, 5.7·10^4)	.901	.901	.384	.538

	Body (CC)	$3.0\cdot10^4$	($-1.7\cdot10^4$, $5.6\cdot10^4$)	.249	.348	$-1.4\cdot10^4$	($-2.5\cdot10^4$, $4.8\cdot10^4$)	.238	.513	.126	.208
	Splenium (CC)	$2.3\cdot10^4$	($-2.1\cdot10^4$, $5.2\cdot10^4$)	.119	.222	$-2.2\cdot10^4$	($-3.8\cdot10^4$, $3.1\cdot10^4$)	.038	.347	.019	.106
	ORs	$3.2\cdot10^4$	($-1.1\cdot10^4$, $6.8\cdot10^4$)	.073	.157	$-1.1\cdot10^4$	($-2.7\cdot10^4$, $5.6\cdot10^4$)	.534	.748	.115	.201
	LGNs	$3.6\cdot10^4$	($-3.4\cdot10^4$, $10.9\cdot10^4$)	.261	.348	$-1.7\cdot10^4$	($-3.4\cdot10^4$, $2.8\cdot10^4$)	.015	.347	.102	.201
	IFOFs	$3.4\cdot10^4$	($-3.3\cdot10^4$, $-7.9\cdot10^4$)	.247	.348	$-2.1\cdot10^4$	($-3.8\cdot10^4$, $5.6\cdot10^4$)	.062	.347	.080	.172
	V1	$6.0\cdot10^4$	($-1.4\cdot10^4$, $13.1\cdot10^4$)	.083	.166	$-2.3\cdot10^4$	($-4.6\cdot10^4$, $4.6\cdot10^4$)	.035	.347	.026	.108
**RNFL thickness (µm)[b]**	Genu (CC)	$-0.023\cdot10^4$	($-1.6\cdot10^4$, $1.3\cdot10^4$)	.970	.970	$0.49\cdot10^4$	($-0.33\cdot10^4$, $1.5\cdot10^4$)	.119	.409	.440	.541
	Body (CC)	$-0.098\cdot10^4$	($-1.7\cdot10^4$, $2.1\cdot10^4$)	.906	.970	$0.31\cdot10^4$	($-0.51\cdot10^4$, $1.3\cdot10^4$)	.146	.409	.615	.689
	Splenium (CC)	$-0.13\cdot10^4$	($-1.9\cdot10^4$, $1.8\cdot10^4$)	.884	.970	$0.61\cdot10^4$	($-0.22\cdot10^4$, $3.1\cdot10^4$)	.141	.409	.464	.541
	ORs	$0.47\cdot10^4$	($-0.93\cdot10^4$, $2.1\cdot10^4$)	.504	.614	$0.54\cdot10^4$	($-0.33\cdot10^4$, $2.1\cdot10^4$)	.114	.409	.923	.923
	LGNs	$-1.9\cdot10^4$	($-3.7\cdot10^4$, $0.21\cdot10^4$)	.043	.105	$0.17\cdot10^4$	($-0.94\cdot10^4$, $0.61\cdot10^4$)	.339	.562	.038	.133
	IFOFs	$-0.50\cdot10^4$	($-2.2\cdot10^4$, $1.1\cdot10^4$)	.483	.614	$-0.14\cdot10^4$	($-1.4\cdot10^4$, $0.41\cdot10^4$)	.611	.778	.439	.541
	V1	$-0.041\cdot10^4$	($-1.9\cdot10^4$, $2.3\cdot10^4$)	.962	.970	$-0.17\cdot10^4$	($-1.8\cdot10^4$, $0.64\cdot10^4$)	.493	.727	.836	.867

BCVA= best corrected visual acuity; CC= corpus callosum; CI= confidence interval; CS= contrast sensitivity; IFOFs= inferior-fronto occipital fasciculus; LGNs= lateral geniculate nucleus; mm^2/s= averaged b-value; ms= milliseconds; ORs= optic radiations; RD= radial diffusivity; p-value^{adj}= Benjamin-Hochberg adjusted p-value; RNFL= retinal nerve fibre layer; ROIs= regions of interest; VLBW= very low birth weight; V1= primary visual cortex; µm= micrometre. [a] Data missing for one VLBW participant and two control participants [b] Data missing for seven VLBW participants and six control participants

Supplemental Table B2. Linear regression with visual outcomes at 32 years as the dependent variable, and group (VLBW versus control) and AD for ROIs (one at a time) at 26 years and their interaction as covariates, adjusting for age and sex

		VLBW (n= 33)				Controls (n= 50)				AD x group	
**Visual outcomes**	**AD in ROIs** (mm^2/s)	**B**	**(95% CI)**	**p-value**	**p-value^{adj}**	**B**	**(95% CI)**	**p-value**	**p-value^{adj}**	***p*-value**	**p-value^{adj}**
**BCVA**	Genu (CC)	$-3.4\cdot10^{4}$	$(-7.7\cdot10^{4}, 0.30\cdot10^{4})$	.117	.688	$-0.29\cdot10^{4}$	$(-2.7\cdot10^{4}, 1.3\cdot10^{4})$	.704	.917	.115	.655
	Body (CC)	$0.04\cdot10^{4}$	$(-2.7\cdot10^{4}, 2.9\cdot10^{4})$	.974	.974	$-0.16\cdot10^{4}$	$(-2.3\cdot10^{4}, 1.3\cdot10^{4})$	.751	.917	.886	.955
	Splenium (CC)	$-0.22\cdot10^{4}$	$(-4.3\cdot10^{4}, 3.0\cdot10^{4})$	.907	.948	$-0.004\cdot10^{4}$	$(-1.9\cdot10^{4}, 1.8\cdot10^{4})$	.995	.997	.910	.955
	ORs	$0.17\cdot10^{4}$	$(-3.3\cdot10^{4}, 2.0\cdot10^{4})$	.879	.948	$-0.05\cdot10^{4}$	$(-1.7\cdot10^{4}, 1.5\cdot10^{4})$	.925	.996	.845	.955
	LGNs	$-1.7\cdot10^{4}$	$(-5.8\cdot10^{4}, 1.6\cdot10^{4})$	.305	.688	$0.31\cdot10^{4}$	$(-1.9\cdot10^{4}, 4.7\cdot10^{4})$	.492	.725	.231	.857
	IFOFs	$-1.2\cdot10^{4}$	$(-5.2\cdot10^{4}, 3.3\cdot10^{4})$	.562	.828	$0.14\cdot10^{4}$	$(-1.8\cdot10^{4}, 2.8\cdot10^{4})$	.763	.917	.504	.955
	V1	$-8.42\cdot10^{4}$	$(-17.3\cdot10^{4}, -0.97\cdot10^{4})$	.054	.625	$0.001\cdot10^{4}$	$(-3.6\cdot10^{4}, 2.9\cdot10^{4})$	.997	.997	.049	.655
**CS function**	Genu (CC)	$-0.30\cdot10^{4}$	$(-0.98\cdot10^{4,} 0.57\cdot10^{4})$	.393	.688	$-0.34\cdot10^{4}$	$(0.79\cdot10^{4}, -0.009\cdot10^{4})$	.059	.380	.921	.955
	Body (CC)	$0.43\cdot10^{4}$	$(-0.41\cdot10^{4}, 1.1\cdot10^{4})$	.339	.688	$-0.33\cdot10^{4}$	$(-1.0\cdot10^{4}, -0.02\cdot10^{4})$	.118	.380	.099	.655
	Splenium (CC)	$-0.05\cdot10^{4}$	$(-0.78\cdot10^{4}, 0.64\cdot10^{4})$	.883	.948	$-0.22\cdot10^{4}$	$(-0.83\cdot10^{4}, 0.07\cdot10^{4})$	.163	.380	.613	.955
	ORs	$-0.49\cdot10^{4}$	$(-1.4\cdot10^{4}, 0.19\cdot10^{4})$	.198	.688	$-0.05\cdot10^{4}$	$(-0.43\cdot10^{4}, 0.72\cdot10^{4})$	.786	.917	.273	.857
	LGNs	$0.26\cdot10^{4}$	$(-0.78\cdot10^{4}, 1.4\cdot10^{4})$	.640	.896	$-0.08\cdot10^{4}$	$(-0.74\cdot10^{4}, 0.98\cdot10^{4})$	.473	.725	.550	.955
	IFOFs	$-0.47\cdot10^{4}$	$(-1.4\cdot10^{4}, 0.33\cdot10^{4})$	.256	.688	$0.12\cdot10^{4}$	$(-0.16\cdot10^{4}, 1.6\cdot10^{4})$	.663	.917	.286	.857
	V1	$-1.4\cdot10^{4}$	$(-3.0\cdot10^{4}, 0.15\cdot10^{4})$	.067	.625	$-0.20\cdot10^{4}$	$(-1.12\cdot10^{4}, 0.50\cdot10^{4})$	.230	.444	.117	.655
**P100 latency (ms)**[a]	Genu (CC)	$1.1\cdot10^{4}$	$(-1.1\cdot10^{4}, 3.8\cdot10^{4})$	.308	.688	$-0.16\cdot10^{4}$	$(-1.7\cdot10^{4}, 3.6\cdot10^{4})$	.894	.996	.447	.955

	Body (CC)	$-1.8\cdot10^4$	($-5.7\cdot10^4$, $2.1\cdot10^4$)	.359	.688	$-1.1\cdot10^4$	($-2.1\cdot10^4$, $1.9\cdot10^4$)	.062	.380	.743	.955
	Splenium (CC)	$-2.0\cdot10^4$	($-4.7\cdot10^4$, $1.5\cdot10^4$)	.179	.688	$-1.5\cdot10^4$	($-3.0\cdot10^4$, $0.57\cdot10^4$)	.109	.380	.729	.955
	ORs	$-1.3\cdot10^4$	($-4.2\cdot10^4$, $3.3\cdot10^4$)	.380	.688	$-1.2\cdot10^4$	($-2.3\cdot10^4$, $2.8\cdot10^4$)	.204	.438	.968	.968
	LGNs	$0.33\cdot10^4$	($-4.3\cdot10^4$, $6.0\cdot10^4$)	.888	.948	$-1.7\cdot10^4$	($-4.1\cdot10^4$, $-0.59\cdot10^4$)	.002	.042	.384	.955
	IFOFs	$0.32\cdot10^4$	($-5.0\cdot10^4$, $5.0\cdot10^4$)	.914	.948	$-2.2\cdot10^4$	($-4.4\cdot10^4$, $-0.92\cdot10^4$)	.003	.042	.306	.857
	V1	$3.9\cdot10^4$	($-2.4\cdot10^4$, $11.1\cdot10^4$)	.214	.688	$-1.6\cdot10^4$	($-2.6\cdot10^4$, $6.0\cdot10^4$)	.157	.380	.115	.655
**RNFL thickness (µm)[b]**	Genu (CC)	$0.17\cdot10^4$	($-0.48\cdot10^4$, $0.82\cdot10^4$)	.554	.828	$0.31\cdot10^4$	($-0.40\cdot10^4$,$0.95\cdot10^4$)	.238	.444	.729	.955
	Body (CC)	$0.067\cdot10^4$	($-1.1\cdot10^4$, $1.3\cdot10^4$)	.896	.948	$0.22\cdot10^4$	($-0.45\cdot10^4$, $0.95\cdot10^4$)	.162	.380	.777	.955
	Splenium (CC)	$0.50\cdot10^4$	($-0.93\cdot10^4$, $1.62\cdot10^4$)	.339	.688	$0.35\cdot10^4$	($-0.18\cdot10^4$, $1.7\cdot10^4$)	.138	.380	.791	.955
	ORs	$0.73\cdot10^4$	($-0.31\cdot10^4$, $1.4\cdot10^4$)	.063	.625	$0.35\cdot10^4$	($-0.29\cdot10^4$, $1.5\cdot10^4$)	.120	.380	.412	.955
	LGNs	$-0.61\cdot10^4$	($-2.1\cdot10^4$, $0.77\cdot10^4$)	.365	.688	$-0.13\cdot10^4$	($-0.80\cdot10^4$, $0.48\cdot10^4$)	.341	.562	.294	.857
	IFOFs	$0.39\cdot10^4$	($-1.2\cdot10^4$, $1.7\cdot10^4$)	.562	.828	$-0.24\cdot10^4$	($-0.51\cdot10^4$, $1.1\cdot10^4$)	.160	.380	.836	.955
	V1	$0.35\cdot10^4$	($-1.4\cdot10^4$, $2.3\cdot10^4$)	.680	.907	$0.21\cdot10^4$	($-1.3\cdot10^4$, $1.2\cdot10^4$)	.269	.471	.877	.955

AD= axial diffusivity; BCVA= best corrected visual acuity; CC= corpus callosum; CI= confidence interval; CS= contrast sensitivity; IFOFs= inferior-fronto occipital fasciculus; LGNs= lateral geniculate nucleus mm^2/s= averaged b-value; ms= milliseconds; ORs= optic radiations; p-value^{adj}= Benjamin-Hochberg adjusted p-value; RNFL= retinal nerve fibre layer; ROIs= regions of interest; VLBW= very low birth weight; V1= primary visual cortex; µm= micrometre. [a] Data missing for one VLBW participant and two control participants [b] Data missing for seven VLBW participants and six control participants

Supplemental Table C1. Mean (SD) of visual outcomes at 32 years and DTI metrics in ROIs at 26 years in the VLBW group and control group, excluding those with NSI

	VLBW (n= 29)		Controls (n= 50)					
	**Mean**	**(SD)**	**Mean**	**(SD)**	**Mean difference**	**95% CI**	**p-value**	**p-value^{adj}**
**Visual outcomes**								
BCVA	87.6	(2.9)	88.2	(4.0)	-0.4	(-1.9, 1.1)	.552	.755
CS function	5.3	(1.6)	6.0	(1.3)	-0.7	(-1.4, -0.03)	.064	.519
P100 latency (ms)[a]	100.3	(5.0)	98.7	(4.7)	1.5	(-0.61, 3.5)	.181	.646
RNFL thickness (µm)[b]	11.7	(1.7)	11.0	(2.3)	0.9	(0.01, 1.7)	.050	.519
**FA/MD**								
FA in genu (CC)	$61.1 \cdot 10^{-2}$	$(2.1 \cdot 10^{-2})$	$61.5 \cdot 10^{-2}$	$(2.3 \cdot 10^{-2})$	$-0.5 \cdot 10^{-2}$	$(-1.5 \cdot 10^{-2}, 0.6 \cdot 10^{-2})$	.340	.754
FA in body (CC)	$61.5 \cdot 10^{-2}$	$(2.6 \cdot 10^{-2})$	$61.9 \cdot 10^{-2}$	$(2.7 \cdot 10^{-2})$	$-0.4 \cdot 10^{-2}$	$(-1.6 \cdot 10^{-2}, 0.8 \cdot 10^{-2})$	.490	.754
FA in splenium (CC)	$73.4 \cdot 10^{-2}$	$(2.6 \cdot 10^{-2})$	$73.0 \cdot 10^{-2}$	$(2.5 \cdot 10^{-2})$	$0.4 \cdot 10^{-2}$	$(-0.8 \cdot 10^{-2}, 1.6 \cdot 10^{-2})$	.514	.754
FA in ORs	$58.6 \cdot 10^{-2}$	$(2.8 \cdot 10^{-2})$	$58.8 \cdot 10^{-2}$	$(3.3 \cdot 10^{-2})$	$-0.4 \cdot 10^{-2}$	$(-1.8 \cdot 10^{-2}, 1.1 \cdot 10^{-2})$	.604	.754
FA in LGNs	$36.1 \cdot 10^{-2}$	$(1.4 \cdot 10^{-2})$	$36.6 \cdot 10^{-2}$	$(1.6 \cdot 10^{-2})$	$-0.5 \cdot 10^{-2}$	$(-1.2 \cdot 10^{-2}, 0.2 \cdot 10^{-2})$	.151	.646
FA in IFOFs	$52.3 \cdot 10^{-2}$	$(2.4 \cdot 10^{-2})$	$52.2 \cdot 10^{-2}$	$(2.8 \cdot 10^{-2})$	$-0.05 \cdot 10^{-2}$	$(-1.3 \cdot 10^{-2}, 1.3 \cdot 10^{-2})$	.943	.943
MD in V1 (mm^2/s)	$7.7 \cdot 10^{-4}$	$(0.32 \cdot 10^{-4})$	$7.6 \cdot 10^{-4}$	$(0.64 \cdot 10^{-4})$	$0.12 \cdot 10^{-4}$	$(-0.12 \cdot 10^{-4}, 0.30 \cdot 10^{-4})$	.315	.754
**AD** (mm^2/s)								
Genu (CC)	$15.4 \cdot 10^{-4}$	$(0.93 \cdot 10^{-4})$	$14.9 \cdot 10^{-4}$	$(1.0 \cdot 10^{-4})$	$0.46 \cdot 10^{-4}$	$(0.093 \cdot 10^{-4}, 0.82 \cdot 10^{-4})$	.034	.519
Body (CC)	$15.7 \cdot 10^{-4}$	$(0.66 \cdot 10^{-4})$	$15.7 \cdot 10^{-4}$	$(1.2 \cdot 10^{-4})$	$0.061 \cdot 10^{-4}$	$(-0.32 \cdot 10^{-4}, 0.39 \cdot 10^{-4})$	.753	.856
Splenium (CC)	$16.3 \cdot 10^{-4}$	$(0.68 \cdot 10^{-4})$	$16.0 \cdot 10^{-4}$	$(1.1 \cdot 10^{-4})$	$0.15 \cdot 10^{-4}$	$(-0.25 \cdot 10^{-4}, 0.49 \cdot 10^{-4})$	.422	.754
ORs	$12.6 \cdot 10^{-4}$	$(0.67 \cdot 10^{-4})$	$12.4 \cdot 10^{-4}$	$(0.89 \cdot 10^{-4})$	$0.25 \cdot 10^{-4}$	$(-0.1 \cdot 10^{-4}, 0.57 \cdot 10^{-4})$	.156	.646
LGNs	$12.3 \cdot 10^{-4}$	$(0.56 \cdot 10^{-4})$	$12.4 \cdot 10^{-4}$	$(1.3 \cdot 10^{-4})$	$-0.11 \cdot 10^{-4}$	$(-0.54 \cdot 10^{-4}, 0.32 \cdot 10^{-4})$	.605	.755
IFOFs	$12.0 \cdot 10^{-4}$	$(0.50 \cdot 10^{-4})$	$11.8 \cdot 10^{-4}$	$(0.89 \cdot 10^{-4})$	$0.13 \cdot 10^{-4}$	$(-0.20 \cdot 10^{-4}, 0.41 \cdot 10^{-4})$	.412	.754
V1	$9.4 \cdot 10^{-4}$	$(0.36 \cdot 10^{-4})$	$9.2 \cdot 10^{-4}$	$(0.72 \cdot 10^{-4})$	$0.16 \cdot 10^{-4}$	$(-0.097 \cdot 10^{-4}, 0.36 \cdot 10^{-4})$	.240	.667
**RD** (mm^2/s)								
Genu (CC)	$5.0 \cdot 10^{-4}$	$(0.59 \cdot 10^{-4})$	$4.8 \cdot 10^{-4}$	$(0.65 \cdot 10^{-4})$	$0.24 \cdot 10^{-4}$	$(-0.01 \cdot 10^{-4}, 0.50 \cdot 10^{-4})$	.083	.519
Body (CC)	$5.0 \cdot 10^{-4}$	$(0.46 \cdot 10^{-4})$	$4.9 \cdot 10^{-4}$	$(0.74 \cdot 10^{-4})$	$0.08 \cdot 10^{-4}$	$(-0.20 \cdot 10^{-4}, 0.32 \cdot 10^{-4})$	.509	.755
Splenium (CC)	$3.7 \cdot 10^{-4}$	$(0.47 \cdot 10^{-4})$	$3.7 \cdot 10^{-4}$	$(0.62 \cdot 10^{-4})$	$-0.025 \cdot 10^{-4}$	$(-0.27 \cdot 10^{-4}, 0.20 \cdot 10^{-4})$	.833	.905
ORs	$4.5 \cdot 10^{-4}$	$(0.47 \cdot 10^{-4})$	$4.4 \cdot 10^{-4}$	$(0.62 \cdot 10^{-4})$	$0.17 \cdot 10^{-4}$	$(-0.08 \cdot 10^{-}, 0.40 \cdot 10^{-})$	.209	.653
LGNs	$7.0 \cdot 10^{-4}$	$(0.36 \cdot 10^{-4})$	$7.1 \cdot 10^{-4}$	$(0.86 \cdot 10^{-4})$	$-0.022 \cdot 10^{-4}$	$(-0.32 \cdot 10^{-4}, 0.19 \cdot 10^{-4})$	.884	.920
IFOFs	$4.9 \cdot 10^{-4}$	$(0.33 \cdot 10^{-4})$	$4.9 \cdot 10^{-4}$	$(0.63 \cdot 10^{-4})$	$0.056 \cdot 10^{-4}$	$(-0.18 \cdot 10^{-4}, 0.25 \cdot 10^{-4})$	.634	.755
V1	$6.9 \cdot 10^{-4}$	$(0.30 \cdot 10^{-4})$	$6.8 \cdot 10^{-4}$	$(0.61 \cdot 10^{-5})$	$0.091 \cdot 10^{-4}$	$(-0.13 \cdot 10^{-4}, 0.26 \cdot 10^{-4})$	.402	.754

Mean difference for participants, adjusted for sex, age at 32 years for visual outcomes and sex and age at 26 years for DTI metrics. AD= axial diffusivity; BCVA= best corrected visual acuity; CC= corpus callosum; CI= confidence interval; CS= contrast sensitivity; FA= fractional anisotropy; IFOFs= inferior-fronto occipital fasciculus; LGNs= lateral geniculate nucleus; MD= mean diffusivity; mm2/s= average b-value; ms= milliseconds; NSI= neurosensory impairments; ORs= optic radiations; p-value^{adj}= Benjamin-Hochberg adjusted p-value; RD= radial diffusivity; RNFL= retinal nerve fibre layer; SD= standard deviation; VLBW= very low birth weight; V1= primary visual cortex; µm= micrometre. [a] Data missing for two control participants [b] Data missing for four VLBW participants and four control participants

Supplemental Table C2. Linear regression with visual outcomes at 32 years as dependent variable, and group (VLBW versus control) and MD for V1 and FA for each remaining ROI (one at a time) and their interaction was entered as covariates, adjusting for age and sex, excluding participants with NSI

		**VLBW – NSI (n= 29)**				**Controls – NSI (n= 50)**				**FA/MD x group**	
**Visual outcomes**	**ROIs**	**B**	**(95% CI)**	**p-value**	**p-value^{adj}**	**B**	**(95% CI)**	**p-value**	**p-value^{adj}**	**p-value**	**p-value^{adj}**
**BCVA**	FA in genu (CC)	33.3	(-30.5, 84.8)	.325	.768	-19.9	(-61.2, 14.6)	.305	.924	.173	.674
	FA in body (CC)	31.4	(-19.9, 79.3)	.242	.678	-9.9	(-51.5, 22.8)	.610	.924	.216	.674
	FA in splenium (CC)	13.2	(-38.5, 64.7)	.634	.893	9.4	(-25.4, 49.1)	.549	.924	.906	.966
	FA in ORs	15.6	(-1.5, 2.3)	.361	.778	10.7	(-16.2, 46.5)	.452	.924	.839	.966
	FA in LGNs	80.1	(6.5, 162.0)	.042	.392	0.92	(-70.4, 69.9)	.977	.996	.141	.674
	FA in IFOFs	24.3	(-9.9, 91.5)	.211	.656	-0.78	(-41.4, 40.2)	.967	.996	.390	.722
	MD in V1 (mm^2/s)	-1.9·10^4	(-6.1·10^4, 1.9·10^4)	.329	.768	0.32·10^4	(-2.4·10^4, 5.9·10^4)	.539	.924	.311	.674
**CS function**	FA in genu (CC)	11.6	(-18.5, 43.7)	.430	.831	-1.5	(-16.1, 9.9)	.822	.996	.439	.722
	FA in body (CC)	21.9	(3.4, 44.5)	.041	.392	1.1	(-12.5, 12.0)	.879	.996	.086	.674
	FA in splenium (CC)	14.6	(-8.2, 31.9)	.151	.532	2.8	(-12.7, 12.5)	.613	.924	.315	.674
	FA in ORs	23.4	(2.6, 46.6)	.032	.392	3.6	(-4.9, 10.8)	.362	.924	.089	.674
	FA in LGNs	16.4	(-16.2, 61.4)	.445	.831	11.4	(-10.7, 34.0)	.326	.924	.842	.966
	FA in IFOFs	24.1	(-5.1, 64.8)	.133	.532	9.3	(-6.7, 26.2)	.232	.924	.420	.723
	MD in V1 (mm^2/s)	-1.0·10^4	(-2.6·10^4, 0.22·10^4)	.144	.532	- 0.21·10^4	(-1.3·10^4, 0.65·10^4)	.200	.924	.272	.674
**P100 latency (ms)**[a]	FA in genu (CC)	-18.0	(-81.2, 81.9)	.647	.893	15.2	(-61.4, 69.3)	.701	.975	.520	.723
	FA in body (CC)	-10.9	(-70.0, 48.2)	.763	.893	15.7	(-52.9, 57.9)	.627	.924	.542	.723
	FA in splenium (CC)	-13.8	(-70.7, 58.1)	.668	.893	35.7	(-40.5, 71.0)	.237	.924	.252	.674
	FA in ORs	-39.4	(-88.5, 20.9)	.152	.532	-1.0	(-60.3, 35.1)	.978	.996	.318	.674
	FA in LGNs	19.1	(-150.5, 178.3)	.797	.893	26.9	(-67.1, 117.5)	.578	.924	.934	.966
	FA in IFOFs	-48.6	(-115.1, 22.5)	.133	.532	9.1	(-70.0, 52.7)	.794	.996	.224	.674
	MD in V1 (mm^2/s)	1.8·10^4	(-6.0·10^4, 8.7·10^4)	.623	.893	-2.0·10^4	(-4.2·10^4, 5.2·10^4)	.054	.924	.321	.674
**RNFL thickness (μm)**[b]	FA in genu (CC)	4.7	(-30.7, 39.7)	.779	.893	--7.8	(-28.3, 15.2)	.438	.924	.529	.723
	FA in body (CC)	4.4	(-19.8, 42.4)	.735	.893	-4.8	(-19.9, 15.02)	.535	.924	.539	.723
	FA in splenium (CC)	7.1	(-22.5, 36.8)	.601	.893	-13.03	(-34.4, 8.9)	.139	.924	.229	.674

FA in ORs	3.0	(-22.4, 24.4)	.796	.893	-9.7	(-23.6, 6.2)	.162	.924	.337	.674
FA in LGNs	-3.4	(-72.4, 97.0)	.911	.976	-8.8	(-60.8, 42.3)	.731	.975	.902	.966
FA in IFOFs	-1.9	(-55.9, 70.0)	.941	.976	-0.19	(-44.7, 53.5)	.996	.996	.966	.966
MD in V1 (mm^2/s)	$0.03 \cdot 10^4$	($-2.1 \cdot 10^4$, $2.3 \cdot 10^4$)	.980	.980	$0.19 \cdot 10^4$	($-1.7 \cdot 10^4$, $1.04 \cdot 10^4$)	.352	.924	.885	.966

BCVA= best corrected visual acuity; CC= corpus callosum; CI= confidence interval; CS= contrast sensitivity; FA= fractional anisotropy; IFOFs= inferior-fronto occipital fasciculus; LGNs= lateral geniculate nucleus; MD= mean diffusivity; mm^2/s= average b-value; ms= milliseconds; NSI= neurosensory impairment; ORs= optic radiations; p-value^{adj}= Benjamin-Hochberg adjusted p-value; RNFL= retinal nerve fibre layer; ROIs= regions of interest; VLBW= very low birth weight; V1= primary visual cortex; µm= micrometre. [a] Data missing for one VLBW participant and two control participants [b] Data missing for seven VLBW participants and six control participants

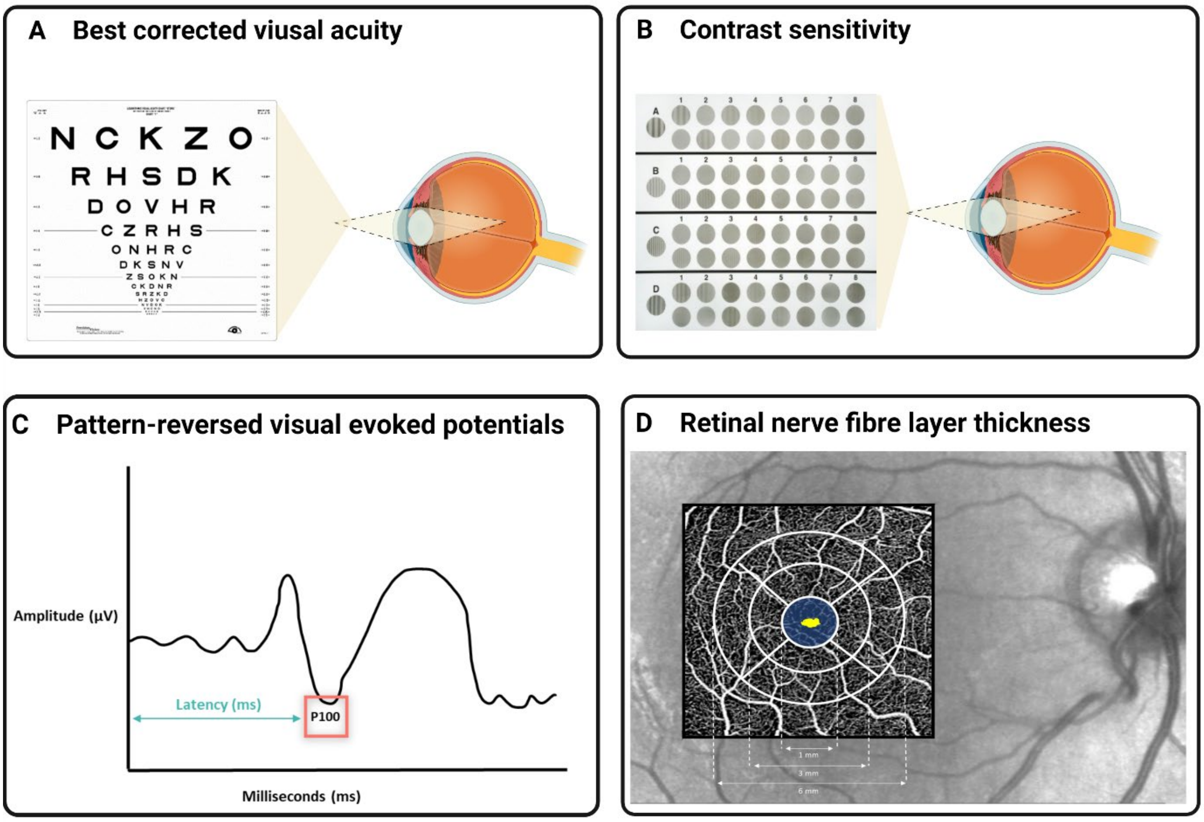

Supplemental Figure A. Illustration of the visual outcomes included in the study. A: BCVA tested with the ETDRS chart where participants are asked to read the letter from the top, how far down they are able to read determines their BCVA ETDRS letter score; a higher score indicates better visual acuity. B: contrast sensitivity testing with the CSV 100-E chart where participants state whether they can see stripes in the circle at the top or the bottom at different contrast levels from 1-8 and spatial frequencies from top to bottom, and your score is determined as how high the contrast level is where you can identify the stripes in the correct circle; a higher contrast level indicates better contrast sensitivity. C: the electrophysiological PR-VEP task where P100 is the first positive peak in electrophysiological activity after stimulus onset (red square), approximately 100 ms after stimulus onset; a P100 latency close to 100 ms indicates normal VEP signaling. D: the ETDRS grid overlayed on an OCT image of the macula where the RNFL thickness is measured in the inner retinal area (marked with blue) which corresponds to the inner ring of 1 mm in the grid; a thinner RNFL in this area indicate more normal retinal architecture.

BCVA= best corrected visual acuity; ETDRS= Early Treatment Diabetic Retinopathy Study; cpd= cycles per degree; mm= milli metre; ms= milliseconds; PR-VEP= pattern-reversed visual evoked potential; RNFL= retinal nerve fibre layer.

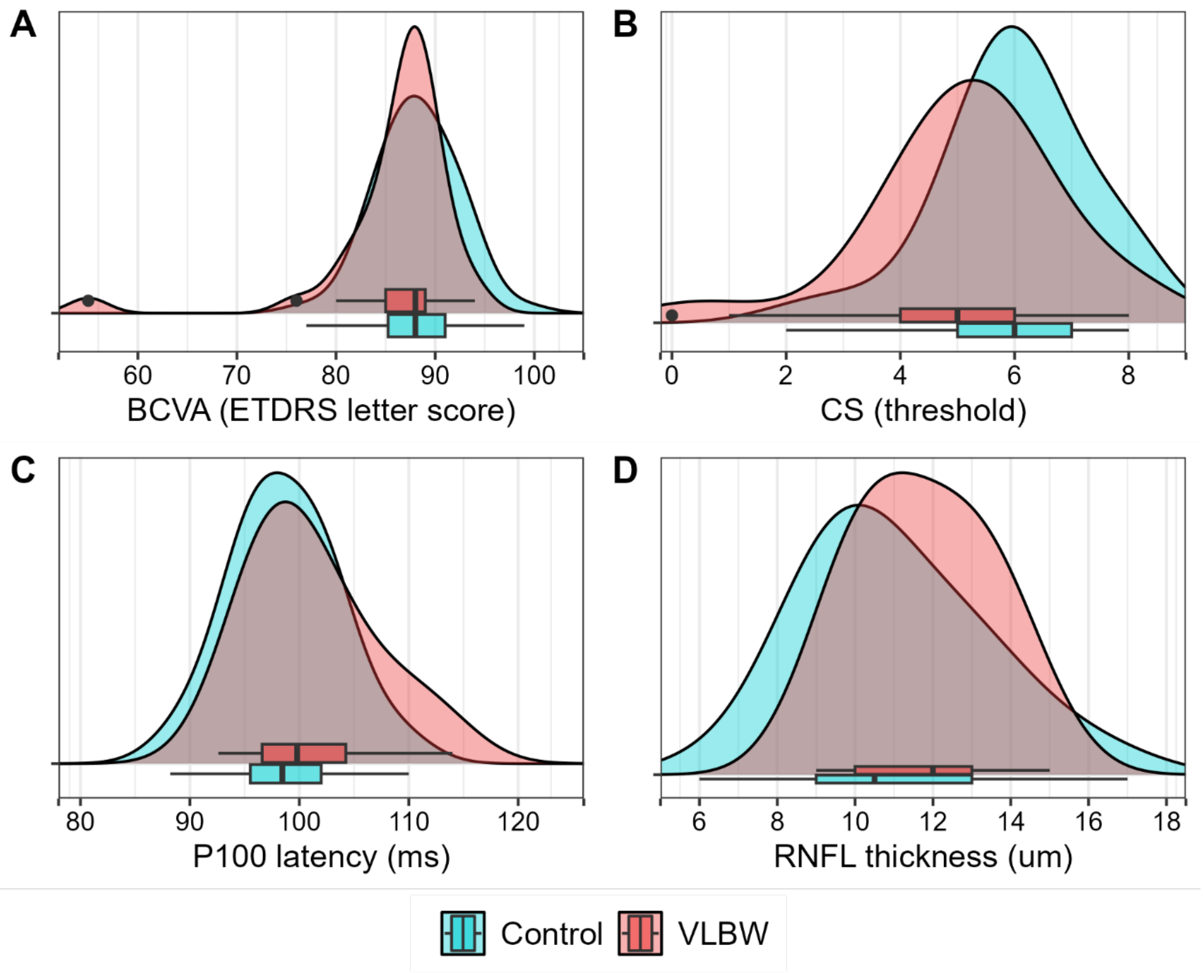

Supplemental Figure B1. Density plots with boxplots of visual outcomes in the control (blue, n=50) and VLBW (red, n=33) group. The y-axis illustrates density estimates, while the x-axis presents the value of the visual outcome, including boxplots with outliers (black circles) below. A= BCVA (0-100, a higher ETDRS letter score represents better visual acuity); B= CS (0-8, a higher threshold indicates better CS); C= P100 latency (a P100 latency closer to 100 ms indicates a visual evoked potential closer to normal); D= RNFL thickness (a larger value/thicker RNFL indicates better conduction of the visual signal).

BCVA= best corrected visual acuity; CS= contrast sensitivity; ETDRS= Early Treatment Diabetic Retinopathy Study; ms= milliseconds; RNFL= retinal nerve fibre layer; µm= micrometre

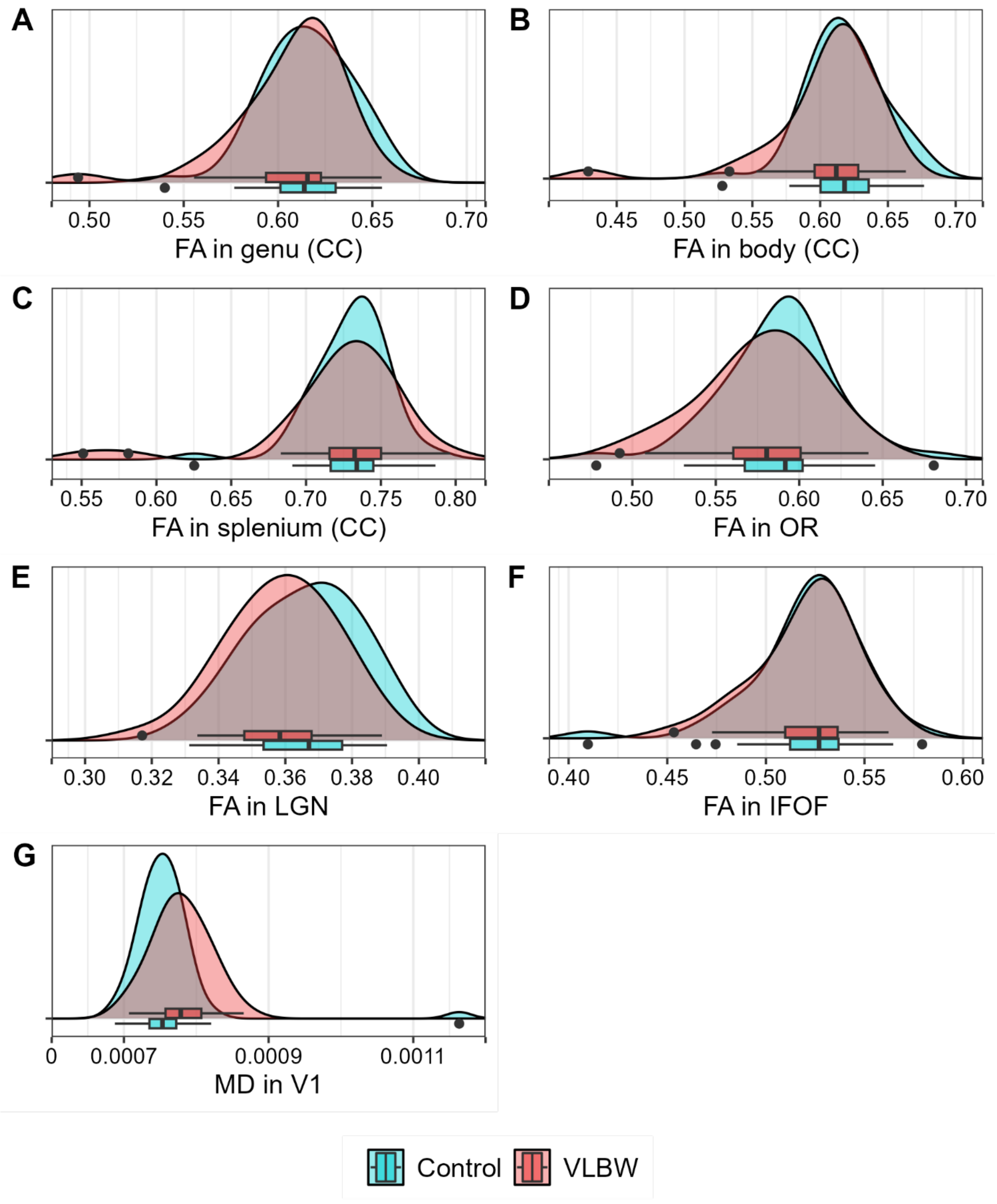

Supplemental Figure B2. Density plots with boxplots of visual outcomes in the control (blue, n=50) and VLBW (red, n=33) group. The y-axis illustrates density estimates, while the x-axis presents FA (0-1, a higher value indicates better white matter integrity) and MD (represents overall diffusivity, a higher value indicates higher diffusivity), including boxplots with outliers (black circles). A= FA in the body of the CC; B= FA in the genu of the CC; C= FA in the splenium of the CC; D= FA in LGN; E= FA in OR; F= FA in IFOF; G= MD in V1.

CC= corpus callosum; FA= fractional anisotropy; IFOF= inferior-fronto occipital fasciculus; LGN= lateral geniculate nucleus; OR= optic radiations; V1= primary visual cortex

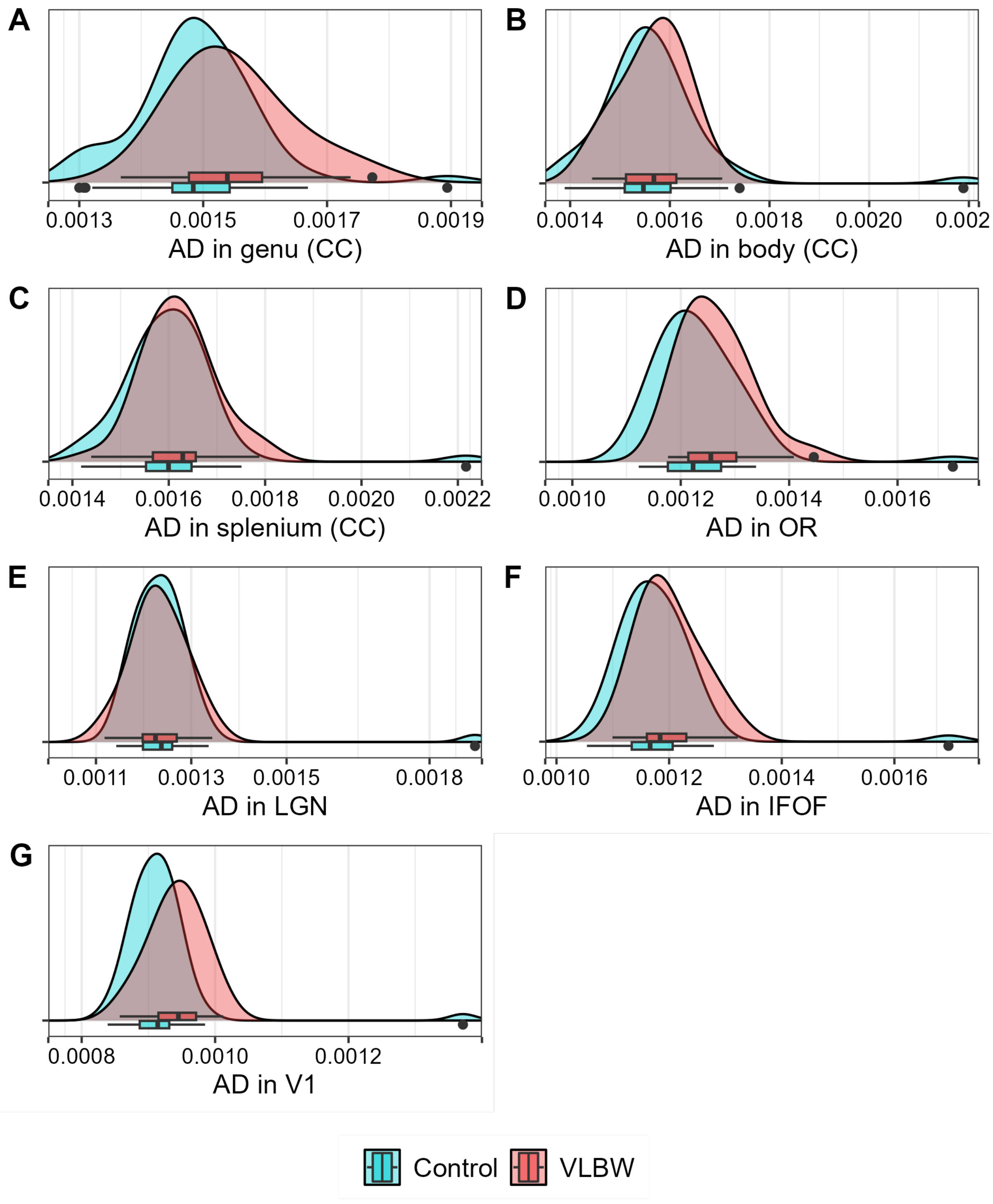

Supplemental Figure B3. Density plots with boxplots of visual outcomes in the control (blue, n=50) and VLBW (red, n=33) group. The y-axis illustrates density estimates, while the x-axis presents AD (represents diffusion parallel to the white matter tracts, a low value indicates axon injury and poor fibre organization), including boxplots with outliers (black circles). A= AD in the body of the CC; B= AD in the genu of the CC; C= AD in the splenium of the CC; D= AD in LGN; E= AD in OR; F= AD in IFOF; G= AD in V1.

AD= axial diffusivity; CC= corpus callosum; IFOF= inferior-fronto occipital fasciculus; LGN= lateral geniculate nucleus; OR= optic radiations; V1= primary visual cortex

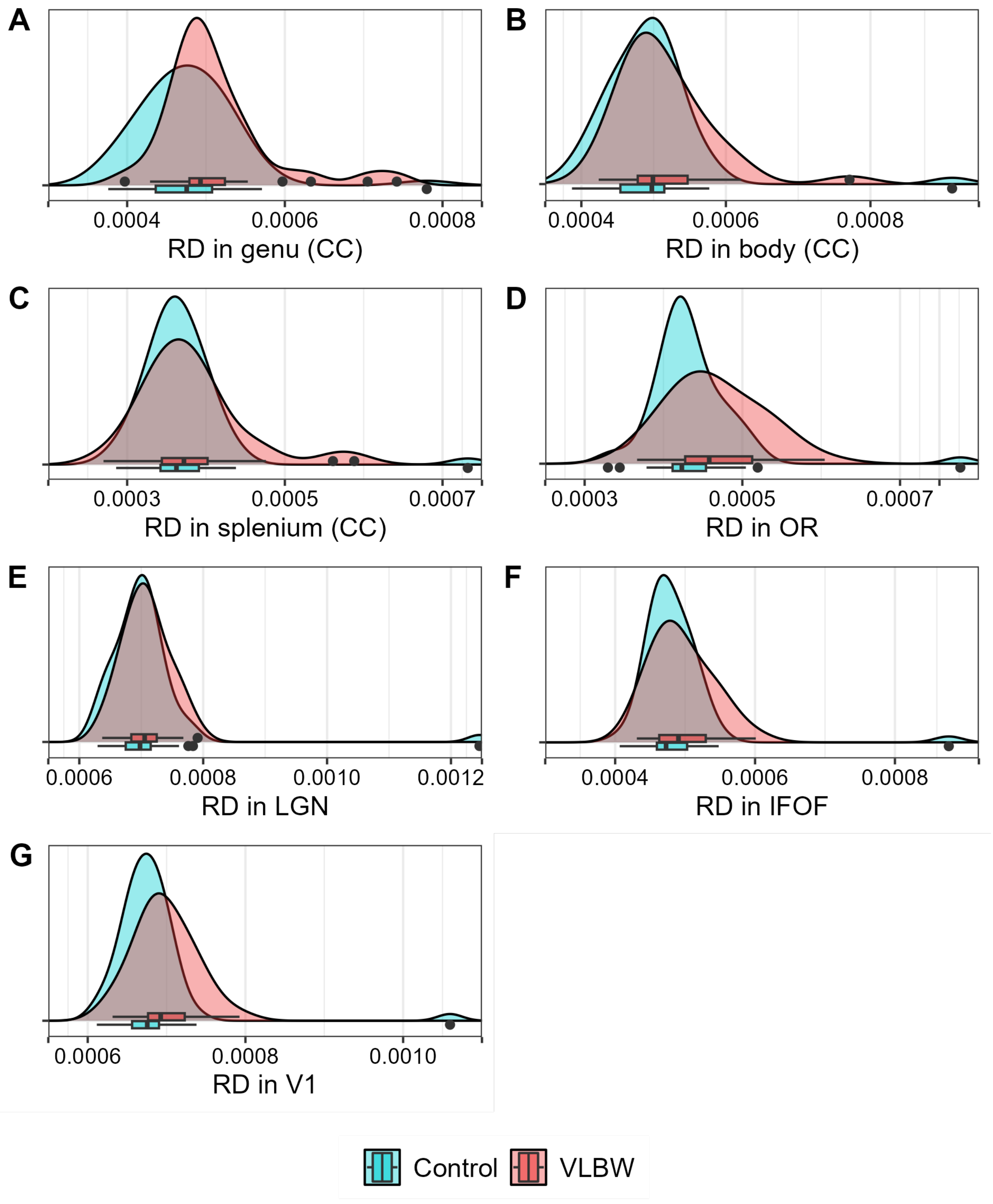

Supplemental Figure B4. Density plots with boxplots of visual outcomes in the control (blue, n=50) and VLBW (red, n=33) group. The y-axis illustrates density estimates. The x-axis presents RD (represents diffusion perpendicular to the white matter tracts where a low value indicates poor myelinization and axon packing). Boxplots with outliers (black circles) are included below. A= RD in the body of the CC; B= RD in the genu of the CC; C= RD in the splenium of the CC; D= RD in LGN; E= RD in OR; F= RD in IFOF; G= RD in V1.

CC= corpus callosum; IFOF= inferior-fronto occipital fasciculus; LGN= lateral geniculate nucleus; OR= optic radiations; RD= radial diffusivity; V1= primary visual cortex
